# Preclinical and Clinical Assessment of Cannabinoids as Anti-Cancer Agents

**DOI:** 10.3389/fphar.2016.00361

**Published:** 2016-10-07

**Authors:** Daniel A. Ladin, Eman Soliman, LaToya Griffin, Rukiyah Van Dross

**Affiliations:** ^1^Department of Pharmacology and Toxicology, Brody School of Medicine, East Carolina UniversityGreenville, NC, USA; ^2^Department of Pharmacology and Toxicology, Faculty of Pharmacy, Zagazig University Zagazig, Egypt; ^3^Center for Health Disparities, East Carolina University Greenville, NC, USA

**Keywords:** cannabinoids, cancer, *in vivo*, clinical studies, clinical trials, therapeutics, cancer models, endocannabinoids

## Abstract

Cancer is the second leading cause of death in the United States with 1.7 million new cases estimated to be diagnosed in 2016. This disease remains a formidable clinical challenge and represents a substantial financial burden to the US health care system. Therefore, research and development of novel therapeutics for the treatment of cancer is of high priority. Cannabinoids and their derivatives have been utilized for their medicinal and therapeutic properties throughout history. Cannabinoid activity is regulated by the endocannabinoid system (ECS), which is comprised of cannabinoid receptors, transporters, and enzymes involved in cannabinoid synthesis and breakdown. More recently, cannabinoids have gained special attention for their role in cancer cell proliferation and death. However, many studies investigated these effects using *in vitro* models which may not adequately mimic tumor growth and metastasis. As such, this article aims to review study results which evaluated effects of cannabinoids from plant, synthetic and endogenous origins on cancer development in preclinical animal models and to examine the current standing of cannabinoids that are being tested in human cancer patients.

## Cannabinoids and cancer

Cannabinoids are a class of hydrophobic molecules that typically bind to G-protein-coupled cannabinoid receptors, cannabinoid receptor type 1 (CB1R) or cannabinoid receptor type 2 (CB2R). Endogenously synthesized cannabinoids (endocannabinoids), cannabinoid receptors, and enzymes involved in cannabinoid formation and breakdown are collectively known as the endocannabinoid system (ECS). This system has been shown to impact different neurological and immunological processes (Zhu, 2006; Tanasescu and Constantinescu, 2010). Additionally, numerous reports demonstrate that endocannabinoids and their putative receptors play a role in the development of various malignancies (Velasco et al., 2016). Many studies investigating the effects of cannabinoids on tumor progression have been carried out in cell culture. However, due to the complexity of the tumor microenvironment, these findings do not always translate to whole animal models. As such, the goal of this review was to compile and analyze the results of studies which evaluated effects of cannabinoids on cancer development in animal models (Table 1) and to examine current knowledge regarding the impact of cannabinoids on tumor growth in humans.

**Table 1:** **Pre-clinical assessment of cannabinoids on tumor development**.

**Cancer type**	**Model type -cell line**	**Animal species/strain**	**Cannabinoid**	**Dose (route)**	**Findings**	**References**
Glioma	Xenograft—C6.9	Immune deficient mice	Delta-9-THC	500 ug/day (p.t.)	Decreased tumor size and tumoral TIMP-1 expression	Blázquez et al., 2008b
			JWH-133	50 ug/day (p.t.)	Decreased tumor size and tumoral TIMP expression, ceramide inhibition increased tumor growth	
	Xenograft—C6	Rag 2-/- mice	WIN 55,212-2	50 ug/day (i.t.)	Decreased tumor size	Galve-Roperh et al., 2000
			Delta-9-THC	500 ug/day (i.t.)		
	Orthotopic—C6	Wistar rat	WIN 55,212-2	50–250 ug/day (i.t.)	Increased survival	
			Delta-9-THC	500–2500 ug/day (i.t.)		
	Xenograft—U87MG	Nude mice	Delta-9-THC	15 mg/kg/day (p.t.)	Decreased tumor size, TUNEL and p8 levels increased in tumor	Salazar et al., 2009
	Orthotopic—U87MG	CB-17 SCID mice	KM-233	2–12 mg/kg twice/day (i.p.)	Decreased tumor size	Gurley et al., 2012
	Patient Derived Xenograft	Athymic Balb/c (nu/nu)	KM-233	12 mg/kg, twice/day (i.p.)	Tumor growth delay	
	Xenograft—C6	Rag 2-/- mice	JWH-133, WIN55,212-2	50 ug/day (i.t.)	Decreased tumor size, effect of JWH-133 prevented by CB2R antagonism	Sánchez et al., 2001
	Xenograft—astrocytoma cells	Rag 2-/- mice	JWH-133	50 ug/day (i.t.)	Decreased tumor size	
	Xenograft—U87MG	Nude mice	Delta-9-THC + TMZ	15 mg/kg THC, 5 mg/kg TMZ (p.t.)	Greater reduction in tumor size than THC or TMZ alone	Torres et al., 2011
			Delta-9-THC + CBD	7.5 mg/kg THC 7.5 mg/kg CBD (p.t.)	Greater reduction in tumor size than THC or CBD alone	
			Delta-9-THC + CBD + TMZ	3.7 mg/kg THC 3.7 mg/kg CBD 5.0 mg/kg TMZ (p.t.)	Greater reduction in tumor size than THC, CBD, or TMZ alone	
	Xenograft—T98G	Nude mice	Delta-9-THC + TMZ	15 mg/kg THC 5.0 mg/kg TMZ (p.t.)	Greater reduction in tumor size than THC or TMZ alone	
	Xenograft—C6.9	Immune deficient mice	Delta-9-THC	500 ug/day (p.t.)	Decreased tumor growth and tumor MMP-2 expression	Blázquez et al., 2008a
			JWH-133	50 ug/day (p.t.)	Decreased tumor size and tumoral MMP-2 expression, ceramide inhibition reduces tumor growth	
	Xenograft—C6	Rag2-/- mice	JWH-133	50 ug/day (i.t.)	Decreased tumor size and tumor expression of VEGFR	Blázquez et al., 2004
	Xenograft—U87MG	Athymic CD1 nude (nu/nu)	CBD	0.5 mg/day (p.t.)	Decreased tumor size	Massi et al., 2004
	Xenograft—C6 cells or primary astrocytoma	Rag2-/- mice	JWH-133	50 ug/day (i.t.)	Decreased tumor size and blood vessel size and functionality	Blázquez et al., 2003
	Orthotopic—U251	Athymic nude (nu/nu)	CBD	15 mg/kg (i.p.)	Decreased tumor size, decreased Id-1 and Ki67 expression in tumor	Aguado et al., 2007
	Xenograft	Athymic nude (nu/nu)	CBD	15 mg/kg (i.p.)	Decreased tumor size, Decreased Id-1 and K167 expression in tumor	Aguado et al., 2007
	Xenograft—U87MG	Nude mice	Delta-9-THC	15 mg/kg/day (p.t.)	Reduced tumor size and increased TRB1, LC3, caspase 3, and decreased S6 in tumors	Salazar et al., 2009
	Xenograft—T98G	Athymic nude	Delta-9-THC	1.5 or 15 mg/kg/day (p.t.)	Decreased tumor size and anti-tumor effect reversed by GRP55 knockdown	Sánchez et al., 2001
	Orthotopic—3832 or 387 cells	Athymic nude (nu/nu)	CBD	15 mg/kg, 5 days/week (i.p.)	Initial decrease in tumor size followed by resistance	Singer et al., 2015
	Orthotopic—GL261	C57Bl/6	CBD + Delta-9-THC	2 mg/kg each (i.p.)	CBD and THC enhanced killing effect of ionizing radiation	Scott et al., 2014
	Xenograft—glioma stem cells	Athymic nude	HU-210, JWH-133	30 uM treatment before inoculation (p.t.)	Decreased tumor growth rate and size decreased stem cell markers increased differentiation markers in tumor	Duntsch et al., 2006
	Xenograft—C6.9	Immune deficient mice	Delta-9-THC	500 ug/day (p.t.)	Decreased tumor size and MMP expression in tumor	Blázquez et al., 2008a
			JWH-133	50 ug/day (p.t.)	Decreased tumor size and MMP expression in tumor, ceramide inhibition prevented tumor regression	
Colon cancer	Azoxymethane-induced colon cancer model	Male ICR mice	CBD	5 mg/kg 3x weekly (i.p.)	Reduced aberrant crypt foci (ACF), number of polyps and tumors	Aviello et al., 2012
	Azoxymethane-induced colon cancer model	Male ICR mice	Cannabis extract rich in CBD	5 mg/kg (i.p.)	Reduced aberrant crypt foci (ACF), number of polyps and tumors	Romano et al., 2014
	Xenograft—HCT-116	Athymic nude female	CBG	3 and 10 mg/kg (i.p.)	Reduced the growth	Borrelli et al., 2014
	Azoxymethane-induced colon cancer models		Male ICR mice	5 mg/kg (i.p.)	Reduced ACF, number of tumor/mouse	
	Xenograft—HT-29	Nude mice	HU-331	5 mg/kg (i.p.)	Reduced angiogenesis	Kogan et al., 2006
	Xenograft—HT-29	Nude mice	HU-331	5 mg/kg (i.p., s.c.,i.t.)	Reduced tumor growth	Kogan et al., 2004
	General *in vivo* toxicity	Sabra male mice, SCID-NOD Mice	HU-331	7.5 mg/kg (i.p.)	Less toxicity than doxorubicin	Kogan et al., 2007
	Xenograft—HT-29		male nude mice	5 mg/kg (i.p.)	Reduced tumor growth and less cardiotoxic than doxorubicin	
	Colitisis induced colon cancer model (Azoxymethane, AOM+dextran sulfate sodium, DSS)	Male CD1 mice	O-1602	3 mg/kg (i.p.)	Reduced number and area of tumors. Decreased histoscore tumor burden and the expression of proliferation marker PCNA. Reduced key mediators that link the inflammation with colorectal cancer such as: inflammatory mediator TNF-α, and oncogenic transcription factors STAT3 and NFκB	Kargl et al., 2013
Liver Cancer	Xenograft—HepG2	Male athymic nude	Delta-9-THC	15 mg/kg (p.t.)	Increased PPAR gamma expression	Vara et al., 2013
			JWH-015	1.5 mg/kg (p.t.)	PPAR-dependent reduction in tumor growth	
	Xenograft—HepG2—and HuH-7	Athymic mice	Delta-9-THC	15 mg/kg (p.t.)	Increase pAMPK, Reduced pAKT, pS6. Autogaphy-dependent reduction in tumor growth	Vara et al., 2011
			JWH-015	1.5 mg/kg (p.t.)		
	Orthotopic—HepG2	Athymic mice	Delta-9-THC	15 mg/kg (i.p.)	Decreased ascites formation, increased pAMPK, Reduced pAKT, pS6. Reduced alpha fetoprotein levels	Vara et al., 2011
			JWH-015	1.5 mg/kg (i.p.)		
	Xenograft—Mz-ChA-1 (cholangiocarcinoma)	Nude mice	AEA	10 mg/kg (i.p.)	GPR55-dependent reduction of tumor growth	Huang et al., 2011b
			O-1602	10 mg/kg (i.p.)		
		Athymic mice	AEA	10 mg/kg (i.p.)	Reduction in tumor growth and VEGF expression	DeMorrow et al., 2008
Pancreatic	Xenograft—MiaPaCa2	Nude mice	Delta-9-THC	15 mg/kg (p.t.)	Reduced tumor growth	Carracedo et al., 2006
			JWH-133	1.5 mg/kg (p.t.)		
	Orthotopic—MiaPaCa2		WIN 55,212-2	1.5 mg/kg (2 days) then 2.25 mg/kg (2 days) then 3.0 mg/kg (10 days) (i.p.)	Reduced the growth and the spreading of pancreatic tumor cells	
Breast	Orthotopic—4T1.2 MVT-1	BALB/c	CBD	10 mg/kg (p.t.)	Decreased tumor growth. Decreased pAkt and EGFR levels	Elbaz et al., 2015
	Orthotopic—MVT-1	FVB				
	Xenograft—MBA-MD-321	Athymic nu/nu	CBD-rich extract	6.5 mg/kg (i.t.)	Decreased tumor growth	Ligresti et al., 2006
	Intraplanar—MBA-MD-321	BALB/c	CBD	5.0 mg/kg (i.p.)	Decreased tumor metastasis	
	Orthotopic—4T1	BALB/c	CBD	1.0, 5.0 mg/kg (i.p.)	Decreased tumor growth and metastasis	McAllister et al., 2011
	Orthotopic—4T1	BALB/c	CBD O-1663	0.5, 1.0, 10 mg/kg CBD; 1.0 mg/kg O-1663 (i.p.)	Decreased tumor growth and metastasis	Murase et al., 2014
	Xenograft—MDA-MB-231	Athymic nu/nu	CBD O-1663	0.5, 1.0, 10 mg/kg CBD; 1.0 mg/kg O-1663 (i.p.)	Decreased tumor metastasis. O-1663 demonstrated greater potency compared to CBD	
	Xenograft—4T1	BALB/c	Delta-9-THC	12.5, 25, 50 mg/kg (s.c.)	Increased tumor growth and Metastasis	McKallip et al., 2005
		SCID-NOD	Delta-9-THC	25 mg/kg (s.c.)	No effect on tumor growth	
	Xenograft (intraplanar)—EMT6	BALB/c	Delta-9-THC	25, 50 mg/kg (i.p.)	No effect on immune response	
	Xenograft (intraplanar)—4T1	BALB/c	Delta-9-THC	25, 50 mg/kg (i.p.)	Decreased anti-tumor immune response in CB2 dependent manner	
	GEMM—MMTV-neu	MMTV-neu	Delta-9-THC	0.5 mg/animal (p.t.)	Decreased tumor growth, multiplicity, and metastasis. Decreased Akt levels	Caffarel et al., 2010
			JWH-133	0.05 mg/animal (p.t.)		
	Xenograft—N202.1	Athymic nu/nu	Delta-9-THC	0.5 mg/animal (p.t.)	Decreased tumor growth is Akt mediated	
			JWH-133	0.05 mg/animal (p.t.)		
	Xenograft—MDA-MB-231	SCID	WIN-55,212-2, JWH-133	5.0 mg/kg (i.p.)	Decreased tumor growth, angiogenesis, and metastasis	Qamri et al., 2009
	GEMM—MMTV-PyMT	MMTV-PyMT	WIN-55,212-2, JWH-133	5.0 mg/kg (i.p.)	Decreased tumor growth and progression	
	GEMM—MMTV-PYMT	MMTV-PyMT	JWH-015	5.0 mg/kg (p.t.)	Decreased tumor volume and weight. Decreased CXCR4 phosphorylation	Nasser et al., 2011
	Orthotopic—NT2.5	FVB				
	Orthotopic—SUM159	Nude Mice	JWH-015	10.0 mg/kg (p.t.)	Decreased tumor volume and weight. Decreased EGFR and IGF-1R signaling	McKallip et al., 2005
	Orthotopic—MCF-7					
	Xenograft—MDA-MB-231	Athymic nu/nu	Synthetic CB2 agonist	2.0 mg/kg (i.p.)	Decrease tumor growth	Morales et al., 2015
	Allograft—TSA-E1	C57BL/6	Met-F-AEA	0.5 mg/kg (i.p.)	Decreased metastasis	Grimaldi et al., 2006
Prostate	Xenograft—LNCaP	MF-1 nude	CBD	1.0, 10, 100 mg/kg (i.p.)	Decreased tumor growth	De et al., 2013
	Xenograft—DU-145	MF-1 nude	CBD	1.0, 10, 100 mg/kg (i.p.)	Potentiated tumor growth	
	Xenograft—PC-3	Athymic nu/nu	JWH-015	0.15 mg/kg (s.c.)	Decreased tumor growth	Olea-Herrero et al., 2009
Lung	Xenograft—A549	NMRI nu/nu	CBD	5.0 mg/kg (s.c.)	Decreased tumor growth, plasminogen Activator inhibitor- 1	Ramer et al., 2010a
	Xenograft—A549	NMRI nu/nu	CBD	5.0 mg/kg (i.p.)	Decreased tumor metastasis	Ramer et al., 2010b
	Xenograft—A549	NMRI nu/nu	CBD	5.0 mg/kg (i.p.)	CBD upregulated ICAM-1 and TIMP-1. ICAM-1 was required for anti-metastatic properties of CBD	Ramer et al., 2012
	Xenograft—A549	NMRI nu/nu	CBD	5.0 mg/kg (i.p.)	CBD decreased tumor growth this was reversed by co-administration with PPAR-γ antagonist	Ramer et al., 2013
	Allograft—LL2	C57BL/6	Delta-9-THC	5.0 mg/kg (p.t.)	No significant effect on tumor growth	McKallip et al., 2005
	Allograft—3LL	C57BL/6	Delta-9-THC	5.0 mg/kg (i.p.)	Increased tumor growth in immunocompetent mode	Zhu et al., 2000
	Xenograft—L1C2	BALB/c	Delta-9-THC	5.0 mg/kg (i.p.)	No effect on tumor growth in immunosuppressed model	
	Xenograft—A549	SCID	Delta-9-THC	5.0 mg/kg (p.t.)	Decreased Tumor growth and metastasis. Decreased Akt	Preet et al., 2008
	Xenograft—3LL	C57BL/6	Met-F-AEA	0.5 mg/kg (i.p.)	Decreased tumor metastasis	Bifulco et al., 2001
	Allograft—3LL	C57BL/6	Met-AEA	5.0 mg/kg (i.p.)	Increased tumor growth in a COX-2 dependent manner	
	Xenograft—L1C2	BALB/c	Met-AEA	5.0 mg/kg (i.p.)	Increased tumor growth in a COX-2 dependent manner	Gardner et al., 2003
Thyroid	Xenograft—KiMol	Athymic Nude	VDM-11	5.0 mg/kg (i.t.)	Decreased tumor size	Bifulco et al., 2004
			AA-5-HT	5.0 mg/kg (i.t.)		
			Met-AEA	0.7 mg/kg (p.t.)	Decreased tumor size, anti-tumor effect blocked by CB1 antagonist	Bifulco et al., 2001
			Met-AEA	0.5 mg/kg (p.t.)	Decreased tumor size, anti-tumor effect blocked by CB1 antagonist, VEGF expression decreased in tumor	
	Xenograft—ARO cells	Balb/c (nu/nu) nude mice	JWH-133	50 ug/ml (i.t.)	CB2 overexpressing ARO cells reduced tumor weight vs. ARO-empty vector cells	
Melanoma	Xenograft—CHL-1	Athymic nude mice	Delta-9-THC	15 mg kg (p.o.)	Reduced tumor growth, Ki67 and increased TUNEL positive cells	Armstrong et al., 2015
	Xenograft—HCmel12 (intracutaneous)	Cnr1/2−/− mice were crossed into the Hgf-Cdk^4*R*24*C*^ melanoma mouse model to generate mice with a dark skin phenotype which develop CB1 and CB2 receptor-deficient melanomas.	Delta-9-THC	5 mg/kg (s.c.)	CB receptors-dependent reduction in tumor growth	Glodde et al., 2015
	Xenograft—B16	C57BL/6 mice and Nude	WIN-55,212–2	50 ug/day (p.t.)	Reduced tumor growth, cell proliferation, apoptosis, and angiogenesis	Blázquez et al., 2006
			JWH-133			
	Xenograft B–16 (intraplantar)	C57BL/6 mice	WIN-55,212–2	50 ug/day (p.t.)	Reduced metastasis in lung and liver	
			JWH-133		JWH-133	
NMSC	Xenograft—PDV.C57	Nude (NMRI nu) mice	WIN-55,212-2	6.7 ug/ul (flow pump—0.52 uL/h (11 days)	Inhibited skin tumor growth, angiogenesis and EGFR activation	Casanova et al., 2003
			JWH-133			
	DMBA/TPA skin carcinogenesis model	Female ICR mice	JWH-018	0.02 and 0.2 uM (t.o.)	Inhibited inflammation, promotion of skin papillomas, tumor incidence	Nakajima et al., 2013
			JWH-122	0.2 and 2 uM (t.o.)		
			JWH-210	0.2 and 2 uM (t.o.)		

*p.t., peritumor; i.t., intratumor; i.p., intraperitoneal; s.c., cubcutaneous; t.o., topical; p.o. oral.*

## *In vivo* animal tumor models

Animal tumor models have become a mainstay in preclinical cancer research due to their ability to produce fairly predictable tumor growth. The first tumor model used to study tumorigenesis was the chemical carcinogenesis model. This model employs the use of two known carcinogens, an initiator and promotor. Initiators are typically employed in a single dose to cause irreversible DNA damage. A promotor is given in repeated doses to increase cell proliferation and induce subsequent DNA damage (Kociba and Schwetz, 1982). Implantable or grafted tumor models are also commonly used. The major subtypes of these models are the (1) syngeneic (allograft) model, in which tumor cell lines of host origin are subcutaneously injected into immune-competent animals and (2) the xenograft model, where cells distinct from the implantation host are subcutaneously injected or grafted into immune-deficient animals (Teicher, 2006). A more technically challenging type of tumor grafting is that of the orthotopic model in which tumor cells or tissues are grafted directly into the organ or system of tumor origin (Peterson and Houghton, 2004). Patient derived xenografts offer high translational value and are created when tumor tissue of human patients is implanted into an immune-compromised animal (Jin et al., 2010). Genetically engineered mouse models (GEMMs) are also used to study carcinogenesis. In the GEMMs, transgenic mice which over-express oncogenes or dominant negative tumor-suppressor genes are often more susceptible to spontaneous tumor induction (Sharpless and Depinho, 2006). Knockout models in which the expression of specific genes is disabled in animals exposed to carcinogens also provide valuable information about mechanisms of tumor formation (Rosenberg et al., 2009). Each of these tumor models is distinct and possess advantages and disadvantages depending upon scientific and clinical goals.

## Brain cancer

It is estimated that more than 23,000 individuals will be newly diagnosed with central nervous system (CNS) cancers in 2016 (Siegel et al., 2016). There are many distinct neuronal and glial tumor types that develop in the brain. Gliomas are a CNS tumor subtype that are derived from glial tissue and account for approximately 80% of all primary malignant brain tumors (Ostrom et al., 2015). The prognosis for this neoplasm is poor with an average 5-year survival rate of 5.1%. As such, novel strategies are needed to impact current therapeutic approaches. The ECS has been examined in clinical and preclinical glioma models. Several studies show that glioma tissues from rodents and humans express functional ECS components including the CB1 and CB2 receptors (Sánchez et al., 2001; Moreno et al., 2014). Furthermore, CB2 expression was found to be elevated in high grade gliomas (Sánchez et al., 2001; Ellert-Miklaszewska et al., 2007) Hence, a series of informative investigations primarily conducted by the Guzman group, have examined the effects of cannabinoids on glioma cell survival with the goal of assessing the feasibility of developing these agents as novel anti-neoplastics.

Numerous studies indicate that Δ^9^-tetrahydrocannabinol (Δ^9^-THC) is a potent inducer of glioma death *in vivo*. Utilizing a C6 glioma cell xenograft model, it was demonstrated that tumor growth was significantly reduced by Δ^9^-THC (Galve-Roperh et al., 2000). Subsequent to orthotopic implantation of the C6 cells the overall survival of Δ^9^-THC treated animals was also increased. *In vitro* studies with the C6 cell line suggested that the Δ^9^-THC-induced reduction in tumor growth occurred as a consequence of increased ceramide synthesis and Erk activation. Another study showed that Δ^9^-THC reduced the growth of U87MG glioma xenografts (Salazar et al., 2009). Experimentation in the U87MG cell line determined that ceramide accumulation and the induction of ER stress proteins p8, CHOP10, and TRB3, were required for Δ^9^-THC-induced death. The authors further demonstrated the importance of p8 in tumor growth by utilizing transformed mouse embryo fibroblasts (MEF) in which tumor development was reduced in p8^+/+^ animals treated with Δ^9^-THC compared to vehicle treated animals. In contrast, no significant difference in tumor growth was observed in Δ^9^-THC or vehicle treated p8^−/−^ mice suggesting that p8 was needed for Δ^9^-THC-mediated tumor death. In a subsequent study by this group, endoplasmic reticulum stress-mediated autophagy and cell death was observed in Δ^9^-THC treated glioma. Specifically, Δ^9^-THC caused an increase in ER stress protein, TRB3, autophagy protein, LC3, and apoptosis as well as a decrease in mTORC protein, p-S6 in U87MG xenograft tumors (Salazar et al., 2009). This pattern of protein expression was also observed in glioma biopsies from 2 patients with glioblastoma multiforme treated via intracranial administration with Δ^9^-THC. *In vitro* experiments with the U87MG cell line suggested that Δ^9^-THC mediated activation of TRB3 was a critical link between the ER stress and autophagy pathways as TRB3 activation was needed for inactivation of Akt and the mTORC1 complex. Other reports indicated that the administration of Δ^9^-THC improves the therapeutic outcome of the clinically utilized glioma anti-neoplastic, temezolamide (TMZ) (Torres et al., 2011). In this study, Δ^9^-THC significantly reduced the growth of U87MG xenograft tumors treated with TMZ when compared to TMZ or Δ^9^-THC alone. Similarly, administration of Δ^9^-THC + cannabidiol (CBD) or Δ^9^-THC + CBD + TMZ significantly reduced tumorigenesis compared to the individual drugs (Torres et al., 2011). The combined drug regimen was associated with prevalent LC3 expression and cell death in U87MG tumors. The mechanism of cell death was also examined in cultured U87MG cells where inhibition of ceramide synthesis and autophagy prevented cellular cytotoxicity (Torres et al., 2011). Since each of these studies also determined that the cytotoxic effect of Δ^9^-THC was dependent on cannabinoid receptors, the findings of these reports indicate that ceramide synthesis, ER stress, autophagy and cannabinoid receptor activation are likely shared mechanism of Δ^9^-THC-induced glioma death.

Cannabinoid regulation of matrix remodeling proteins may also play a role in its anti-tumor activity. Matrix metalloproteinases (MMP) can promote or prevent tumor invasion and metastasis by degrading extracellular matrix and other cell-associated proteins (Lukaszewicz-Zajac et al., 2014). In addition, the effects of MMPs are counteracted by tissue inhibitors of metalloproteinases (TIMPs). MMPs/TIMPs also directly regulate tumor growth, tumor apoptosis and angiogenesis. An examination of the effect of Δ^9^-THC on C6.9 cell xenograft growth showed that animals treated with Δ^9^-THC contained smaller tumors with reduced MMP-2 expression in contrast control group animals (Blázquez et al., 2008a). Δ^9^-THC also reduced tumor growth and the expression of MMP-2 in tumors of Δ^9^-THC-treated patients. Cell culture studies with C6.9 cells confirmed that MMP-2 expression and cell viability were decreased as a result of Δ^9^-THC exposure (Blázquez et al., 2008a). A different study by this research group also indicated selective participation of TIMP-1 in the anti-neoplastic activity of Δ^9^-THC (Blázquez et al., 2008b). Similar to MMPs, TIMPs have both a positive and negative impact on tumor growth (Lukaszewicz-Zajac et al., 2014). In C6.9 xenografts, Δ^9^-THC decreased tumor development and tumoral TIMP-1 but had little impact on TIMP-2 or TIMP-3 expression. Selective TIMP-1 downregulation was also observed in patient tumor samples subsequent to Δ^9^-THC administration. C6.9 cell culture studies showed that Δ^9^-THC decreased TIMP-1 expression by increasing ER stress and ceramide synthesis and also demonstrated that Δ^9^-THC reduced C6.9 cell migration (Lukaszewicz-Zajac et al., 2014). These studies suggest that downregulation of MMP-2 and TIMP-1 is important for Δ^9^-THC regulation of tumor size, however additional work is needed to reveal whether the reduction in these matrix modulating proteins also alters the metastatic and invasive behavior of tumor cells *in vivo*.

An investigation of the effects of Δ^9^-THC on human glioblastoma-T98G xenografts demonstrated concentration-dependent effects with low Δ^9^-THC concentrations causing a modest increase in tumor growth and high Δ^9^-THC concentrations promoting tumor cell death (Sánchez et al., 2001). Experiments in cultured T98G cells suggested that this effect was mediated by the formation of a functional dimeric complex between GPR55 and CB2 receptors such that low concentrations of Δ^9^-THC activated the CB2 receptor and Erk expression thereby promoting tumor cell growth. However, in the presence of high Δ^9^-THC concentrations, Δ^9^-THC inhibited GPR55 which blocked the CB2 receptor and Erk activation thereby inhibiting tumor cell growth. This finding is consistent with reports showing that the putative G-protein-coupled cannabinoid receptor, GPR55, is overexpressed in cancers and promotes carcinogenesis (Falasca and Ferro, 2016).

Most clinically available cancer chemotherapeutic agents demonstrate significant toxicity toward cancer and normal cells. This lack of selectivity often produces undesirable side effects that limit its therapeutic utility. Δ^9^-THC preferentially induced cell death in tumorigenic compared to non-tumorigenic astrocytes (Salazar et al., 2009). Moreover, in non-tumor bearing rats, Δ^9^-THC (2500 μg) exhibited limited toxicity and did not affect animal survival, health, behavior or hematopoietic parameters (Galve-Roperh et al., 2000). These findings suggest that Δ^9^-THC will cause limited adverse effects thereby making this compound an attractive candidate for therapeutic use. Furthermore, the anti-emetic, appetite stimulating and analgesic effects of Δ^9^-THC and other cannabinoids can provide relief for important co-morbidities commonly associated with cancer and cancer chemotherapy.

Cannabidiol (CBD) is a non-psychotropic phytocannabinoid whose effect on tumor growth is being extensively explored. CBD significantly reduced U87MG xenograft growth when compared to vehicle control animals (Massi et al., 2004). An *in vitro* examination of the mechanism of action suggested that the induction of reactive oxygen species (ROS) and activation of the CB2 receptor was involved in the anti-tumor effect. However, unlike Δ^9^-THC, CBD toxicity did not appear to be regulated by CB1 or ceramide. Furthermore, in an orthotopic and subcutaneous tumor model, CBD significantly decreased U251 cell tumor growth (Soroceanu et al., 2013). In tumors generated by both methods, the expression of the proliferation marker, Ki67, and inhibitor of DNA binding (Id-1) protein were reduced. Id-1 is a transcription factor that prevents tumor cell differentiation and its expression is elevated in highly aggressive tumors (Ling et al., 2006). Cell culture studies showed that CBD inhibited Id-1 expression which occurred coincident with a reduction in U251 cell invasiveness (Soroceanu et al., 2013). A follow up study by this group demonstrated that CBD inhibited tumor growth and increased the survival of animals orthotopically implanted with primary 3832 and 387 glioblastoma cells (Singer et al., 2015). The reduction in tumor size was accompanied by decreased expression of Id-1 and Sox-2 indicating a reduction in tumor aggressiveness. Continued administration of CBD in this model resulted in reduced responsiveness to CBD, increased antioxidant response gene expression and upregulated expression of mesenchymal markers in the tumors. Investigation of the *in vitro* mechanism suggested that resistance to CBD was primarily caused by activation of SCL7A11 (a transcriptional target of Nrf2) antioxidant signaling as inhibition of this target decreased tumor cell survival and augmented the antitumor activity of CBD. A different study revealed that CBD potentiated the killing effect of ionizing radiation (IR), a common clinical therapy for glioblastoma. Using an orthotopic xenograft model, CBD + Δ^9^-THC + IR significantly decreased the size of GL261 gliomas compared to CBD + Δ^9^-THC or IR alone (Scott et al., 2014). Increased TUNEL positive cells in tumors isolated from animals exposed to CBD + Δ^9^-THC + IR implicated cell death as the mechanism of tumor growth inhibition. Thus, the antitumor activity of Δ^9^-THC and CBD has been demonstrated in different *in vivo* tumor models and the diverse pathways by which these agents elicit death appear to favor co-administration with other chemotherapeutic agents.

Numerous studies suggest that synthetic cannabinoids such as JWH-133, WIN 55,212-2 and HU-210 are promising chemotherapeutic agents for treatment of gliomas. In murine C6 xenografts, the growth of glioma solid tumors was significantly inhibited by JWH-133 compared to control group animals (Sánchez et al., 2001). This effect was found to be mediated by the CB2 but not the CB1 receptor. In the same animal model, JWH-133 also inhibited the growth of human glioma cells. Examination of the mechanism of cytotoxicity in cultured C6 cells showed that ceramide synthesis was needed for JWH-133-mediated tumor cell death. Later studies by this group validated the *in vitro* mechanism by demonstrating that blockade of ceramide synthesis *in vivo* decreased the anti-tumor activity of JWH-133 (Blázquez et al., 2008a,b). These studies also revealed that ceramide was involved in JWH-133-induced suppression of the invasion associated proteins, MMP-2 and TIMP-1. The combined reports suggest that the induction of ceramide synthesis by JWH-133 causes a reduction tumor cell survival and invasion.

The effect of synthetic cannabinoids on angiogenesis was also examined. JWH-133 reduced the growth of C6 gliomas that was accompanied by a decrease in tumoral, pro-angiogenic VEGF and VEGFR (Blázquez et al., 2004). It was determined that VEGFR levels were also downregulated in glioma biopsies from patients treated with Δ^9^-THC. *In vitro* studies with C6 cells demonstrated that both JWH-133 and WIN 55,212-2 decreased VEGFR2 phosphorylation and VEGF levels by increasing ceramide production. The reduction in VEGF signaling by the cannabinoids was also dependent on CB1 and CB2 receptors. A more detailed examination of the effect of cannabinoids on angiogenesis showed that JWH-133 decreased the permeability of tumor localized blood vessels as well as the growth of C6 and patient-derived glioma cells in xenograft tumor studies (Blázquez et al., 2003). Consistent with these results, the mRNA expression of the angiogenesis and metastasis-promoting proteins VEGF, Ang2 and MMP-2 were decreased by JWH-133. Furthermore, *in vitro* studies using HUVEC cells demonstrated that JWH-133 inhibited endothelial cell migration and initiated death that was reversed by antagonism of CB1 and CB2 receptors. The findings of these reports indicate that cannabinoid prevention of angiogenesis is reliant on ceramide production and cannabinoid receptor activation.

Accumulating evidence suggests that a subpopulation of brain tumors originate from pluripotent stem-like cells (Singh et al., 2004). These undifferentiated cells are often associated with poor prognosis and highly aggressive tumor phenotypes whereas well-differentiated glial cells are typically less aggressive (Wan et al., 2011). As such, the stem-like population of U87MG cells and human gliomas were treated with JWH-133 or HU-210 and then subcutaneously implanted to examine the effect of cannabinoids on this tumor cell subtype (Aguado et al., 2007). Cannabinoid treated cells formed smaller tumors which contained low levels of stem-like stage proteins such as nestin and high levels of differentiation-associated proteins including β-tubulin III and S-100β. Consistent with these findings, JWH-133 and HU-210 decreased the expression of nestin and increased the expression of GFAP, S-100β and β-tubulin III in cultured stem-like glioma cells. Hence it appears that the anti-neoplastic activity of the synthetic cannabinoids may be attributed to their ability to prevent tumor cell survival, invasion, and angiogenesis while promoting tumor cell differentiation.

KM-233 is a synthetic cannabinoid that signals through CB1 and CB2 receptors and is a derivative of Δ^8^-THC (Krishnamurthy et al., 2008). The anti-neoplastic activity of KM-233 was examined in an orthotopic U87MG cell tumor protocol where a significant reduction in tumor size occurred (Gurley et al., 2012). These results were verified in a xenograft tumor study using patient-derived glioblastoma multiforme tissue. *In vitro* studies showed that KM-233 induced U87MG death was prevented by antagonizing the CB1 receptor. A subsequent study by this group confirmed the anti-tumor activity of KM-233 against U87MG xenograft tumors and also demonstrated that KM-233 produced minimal damage to neuronal tissue within the therapeutic dosage range (Duntsch et al., 2006). As such, KM-233 may be an effective agent against glioma with an acceptable safety profile similar to other cannabinoids.

The bulk of our knowledge about the effect of cannabinoids on cancer growth in *in vivo* models is derived from the aforementioned glioma studies. Much of this work demonstrated potent antitumor activity, confirmed *in vitro* mechanisms of cannabinoid antitumor action and provided evidence that these molecules were minimally toxic and therefore paved the way for execution of a clinical study with a small number of glioma patients (Clinical trial ID# NCT01812603). However, additional experimentation in animals is needed to define the pharmacokinetic properties of cannabinoids before large scale human studies are conducted. It will be particularly important to examine drug accumulation in tumors and drug disposition in body compartments subsequent to drug administration via different routes as most of the glioma studies conducted thus far employ intra/peri-tumor drug delivery. Such investigations may identify more clinically convenient routes of administration, establish the extent of drug stability and metabolism while providing clues about potential adverse effects of these agents.

## Digestive system cancer

Digestive system cancers include colon, stomach, liver, and pancreatic cancer. In the United States, these combined malignancies account for the highest incidence of cancer besides skin cancer and are the second leading cause of death for both sexes. In 2016, an estimated 304,930 new cases of digestive system cancer will be diagnosed in the US with 153,030 estimated deaths (Siegel et al., 2016).

### Colorectal cancer

Colorectal cancer (CRC) is the most lethal type of digestive cancer followed by pancreatic and then liver cancer. Recent studies advocate that the ECS plays a critical role in CRC development and is therefore considered an appropriate target for CRC inhibition. The levels of the endocannabinoid, anandamide (AEA), the enzymes participating in AEA biosynthesis and degradation, as well as its molecular target CB2, are elevated in tumor specimens of CRC patients (Cianchi et al., 2008; Chen et al., 2015). Furthermore, there was a positive correlation between CB2 receptor expression and human colon cancer growth. Consistent with this finding, the CB2 receptor was a poor prognostic marker in advanced stages of colon cancer (stage III and IV) (Martinez-Martinez et al., 2015). Hence, the ECS may significantly impact colon tumor progression. The anti-cancer activities of phyto- and synthetic cannabinoids in colon cancer animal models have also been reported. In chemically-induced azoxymethane (AOM) colon carcinogenesis, the phytocannabinoids, CBD and Cannabis sativa extract (which contains high CBD content, one of the main components of the botanical drug, Nabiximols) reduced aberrant crypt foci (ACF) formation and the number of precancerous polyps and tumors (Aviello et al., 2012; Romano et al., 2014). *In vitro* experiments in the same studies suggested that the cytotoxicity was mediated by CB1, TRPV1, and PPARγ (CBD and Cannabis extract) or CB2 (Cannabis extract) (Aviello et al., 2012; Romano et al., 2014). Furthermore, the non-psychoactive phytocannabinoid, cannabigerol (CBG), reduced the growth and development of HCT-116 xenografts and AOM-induced colon cancer formation. Using the HCT-116 cell lines, cell death was prevented by blocking TRPM8 but not CB1, CB2, or TRPV1 receptors (Borrelli et al., 2014). Synthetic cannabinoids also showed anti-cancer activity in different animal models. HU-331, a quinone compound synthesized from cannabidiol, reduced angiogenesis and tumor growth in a HT-29 xenograft model (Kogan et al., 2004, 2006). In nude mice xenografts with HT-29 cells, HU-331 also showed greater inhibition of tumor growth than the clinically utilized chemotherapeutic, doxorubicin (Kogan et al., 2007). Moreover, a comparative study utilizing multiple animal models showed that HU-331 exhibited less cardiotoxicity than doxorubicin. In a different study, O-1662 an analog of abnormal cannabidiol, reduced the number and size of tumors formed by AOM + dextran sulfate sodium in the colitis-induced colon cancer model. This activity was accompanied by reduced inflammation, proliferation, and induction of apoptosis in the tumor tissue (Kargl et al., 2013). Although numerous studies tested the effect of cannabinoids and their derivatives in different CRC models, reports that utilize agents which bind selectively to CB1 or CB2 receptors are lacking. As such, the direct role of cannabinoid receptor activation in the context of CRC treatment has not been addressed *in vivo*.

### Liver cancer

There are two major types of liver cancer, hepatocellular carcinoma (HCC; also known as hepatoma), which originates in hepatocytes and accounts for approximately 75% of liver cancers and cholangiocarcinoma, which develops within the bile ducts of the liver (Huang et al., 2011a). Xu et al. found that the expression of both CB1 and CB2 receptors was increased in HCC tissues and was strongly associated with improved prognosis and disease-free survival rates (Xu et al., 2006). Conversely, Mukhopadhyay et al. reported that CB1 receptors promote the initiation and progression of diethylnitrosamine (DEN)-induced HCC in mice (Mukhopadhyay et al., 2015). Hence, it is appropriate to investigate whether cannabinoid receptors are suitable targets for treatment of liver cancer. Phytocannabinoid, Δ^9^-THC, and synthetic CB2 receptor agonist, JWH-015 reduced the growth of HepG2 and HuH-7-derived tumor xenografts and diminished ascites development in an orthotopic model of HCC. The anti-tumor activities of Δ^9^-THC and JWH-015 in hepatocellular carcinoma cell lines (HepG2 and HuH-7) were mediated by the activation of CB2 receptors followed by increased ceramide, ER-stress, PPAR-γ activity, and eventually the induction of autophagy (Vara et al., 2011, 2013). Furthermore, the endocannabinoid, AEA, reduced the growth of Mz-ChA-1-derived cholangiocarcinoma xenografts and downregulated the tumor expression of angiogenic factors, VEGF-C, VEGF-R2, and VEGF–R3 in AEA-treated tumors. The anti-neoplastic activity of AEA was mediated by GPR55 receptors (*in vivo* and *in vitro*), activation of Wnt/JNK signaling (*in vitro*), and recruitment of the Fas death receptor into lipid rafts (*in vitro*) (DeMorrow et al., 2008; Huang et al., 2011b). These studies provide strong support that phyto, synthetic, and endogenous cannabinoids confer anti-proliferative effects on liver tumor cells, however, in these reports xenografts are the primary model utilized. Additional insight would be gained by assessing the effect of cannabinoids on HCC using chemical carcinogenesis models which evaluate tumor growth with clinically relevant carcinogens in their native microenvironment.

### Pancreatic cancer

The ECS plays an active role in pancreatic carcinogenesis. It was reported that CB1 and CB2 receptor expression was elevated in human pancreatic tumors when compared to normal pancreas (Carracedo et al., 2006; Michalski et al., 2008). Although the levels of endocannabinoids, AEA, 1-AG, and 2-AG were unchanged in pancreatic cancer compared to normal human pancreas, high levels of CB1 receptor expression and low levels of endocannabinoid degrading enzymes, FAAH and monoacyl glycerol lipase (MAGL), were associated with shorter survival (Michalski et al., 2008). On the other hand, Carracedo et al., demonstrated that Δ^9^-THC and JWH-133 reduced growth of MiaPaCa2-derived xenografts. Additionally, WIN-55,212-2 reduced the growth of orthotopically implanted MiaPaCa2 pancreatic tumors and prevented the spread of tumor cells to distal organs (Carracedo et al., 2006). *In vitro* studies showed that the anti-proliferative activity of the cannabinoids was mediated by the activation of CB2 receptors followed by *de novo* synthesis of ceramide and the induction of ER-stress (Carracedo et al., 2006).

Collectively, the ECS plays an important role in the progression of digestive cancers including colon, liver, and pancreatic cancers. Cannabinoid receptor expression is elevated in tumors when compared to normal tissues, indicating that endogenous cannabinoids have cancer promoting activity. However, preclinical studies revealed that exogenous synthetic, phyto-, and endo- cannabinoids reduced tumor incidence, growth, and angiogenesis. Although sufficient evidence is not available to explain this effect, these seemingly opposing events may be explained by the biphasic effect of cannabinoids. Whereas low cannabinoid concentrations (<10 uM) increase tumor progression, high concentrations (>10 uM) reduce cancer cell proliferation (Martinez-Martinez et al., 2015). Further research is required to uncover potential *in vivo* biphasic effects and determine the optimum drug concentrations needed to prevent pancreatic cancer growth. Moreover, since the number of pancreatic tumor studies with cannabinoids is extremely limited, more research is required to demonstrate cannabinoid efficacy.

## Breast cancer

Breast cancer is the most commonly diagnosed cancer in women and the second leading cause of female cancer deaths in the United States (Siegel et al., 2016). Risk factors for developing this type of cancer include: being of the female sex, having a family history of this disease, obesity, and menopause-associated hormone replacement (Kaminska et al., 2015). Recent research suggested that cannabinoids and the ECS played a role in breast cancer progression and may possess therapeutic potential (Caffarel et al., 2012).

Breast cancer can be divided into three sub-types: hormone sensitive breast cancer, which expresses estrogen receptors (ER) and/or progesterone receptors (PR); HER2-positive breast cancer, which expresses human epidermal growth factor receptor (ErbB2, a member of the EGFR family); and triple negative breast cancer (TNBC), a type of breast cancer which is difficult to treat due to the lack of ER, PR, and HER2 expression. Cannabinoid receptor expression differs between these breast cancer sub-types. CB1 receptors were reported to be present in 28% of all breast carcinoma patients, with 14% being HER2 positive. On the other hand CB2 receptors were found in 72% of breast carcinomas and 91% of these tumors were HER2 positive, suggesting a possible link between HER2 and CB2 expression (Qamri et al., 2009; Caffarel et al., 2010; Pérez-Gómez et al., 2015). Of interest, a correlation between CB2 receptor expression and breast cancer aggressiveness has been suggested (Caffarel et al., 2006). As such, the effects of cannabinoids on breast tumor growth and metastasis using *in vivo* models has helped elucidate their therapeutic and prognostic potential.

Phytocannabinoids such as Δ^9^-THC and CBD induce both breast cancer regression and progression *in vivo*, indicating that a complex interaction between these compounds and cancer exists. CBD was shown to decrease tumor growth in multiple breast cancer models including: two orthotopic models, one genetically engineered mouse model (GEMM) and one TNBC xenograft model (Ligresti et al., 2006; McAllister et al., 2011; Murase et al., 2014; Elbaz et al., 2015). CBD significantly decreased the incidence of lung metastasis in TNBC xenograft metastatic and orthotopic mouse models. Furthermore, two of these studies which used genetically engineered and xenograft models proposed that CBD elicited these effects by inhibiting EGFR activation, cytokine secretion, and Akt expression (McAllister et al., 2011; Elbaz et al., 2015). The CBD derivative, O-1663, was also shown to decrease TNBC xenograft growth through a mechanism requiring the generation of oxidative stress (Murase et al., 2014). Consistent with the findings for CBD, Δ^9^-THC impaired tumor growth, multiplicity and metastasis in both HER2 positive MMTV-neu mice (a GEMM) and HER2 positive xenografted mice (Caffarel et al., 2010). This group also found that downregulation of pro-tumorigenic Akt was involved in the anti-proliferative effects of Δ^9^-THC (Caffarel et al., 2010). In contrast, phytocannabinoids also promoted tumor development. The Nagarkatti group found that mice implanted with 4T1 cells that were treated with Δ^9^-THC displayed greater tumorigenicity and metastatic potential by inhibiting host anti-tumor immune responses (McKallip et al., 2005). Indeed, phytocannabinoid-induced immune suppression has been reported to be mediated by the CB2 receptor in similar tumor models (Zhu et al., 2000).

Synthetic cannabinoid receptor agonists have also been utilized to elucidate the role of CB1 and CB2 receptors in breast cancer growth. Activation of CB1/CB2 receptors using WIN-55,212-2 led to significant decreases in tumor growth and lung metastasis in a TNBC xenograft and a genetically engineered MMTV-PyTV (mouse mammary tumor virus encoding polyoma virus middle T antigen) mouse model (Qamri et al., 2009). Furthermore, selective CB2 receptor activation with JWH-133 inhibited the growth of tumors and decreased metastasis in MMTV-PyTV mice, HER2 positive MMTV-neu mice and MDA-MB231-derived xenografts (Qamri et al., 2009; Caffarel et al., 2010; Morales et al., 2015). Follow-up *in vitro* studies performed in MDA-MB231, MDA-MB468 and N202.1 cells also suggested that CB2 receptor activation by synthetic cannabinoids such as JWH-133 downregulated pro-tumorigenic prostaglandin E_2_ (PGE_2_) or Akt similar to phytocannabinoids (Qamri et al., 2009; Caffarel et al., 2010). Another CB2 receptor agonist, JWH-105, was found to inhibit tumor growth in a NT2.5-orthotopic mouse model and the MMTV-PyMT model and this observation corresponded to decreased pro-metastatic phospho-CXCR4 and phospho-ERK levels *in vivo* and *in vitro* (Nasser et al., 2011). Additionally, activation of CB2 receptors by a similar compound, JWH-015, was also found to decrease tumor growth and weight in both, ER- positive and ER- negative orthotopic models (Elbaz et al., 2016). JWH-015 treated mice exhibited decreased EGFR and IGF-1R signaling and Akt expression (Elbaz et al., 2016). Finally, the synthetic and metabolically stable analog of AEA, 2-methyl-2'F-anandamide (Met-F-AEA), was also shown to decrease lung metastasis in TSA-E1 cell-derived xenografts (Grimaldi et al., 2006). For the most part, *in vivo* studies indicate that phyto-, synthetic- and endo- cannabinoids suppress tumor development in hormone receptor positive, EGFR positive and triple negative breast cancer animal models. However, additional animal studies are needed to understand how cannabinoid regulation of the immune system impacts breast tumor growth.

## Prostate cancer

Carcinoma of the prostate is the development of malignant cells within the male reproductive prostate gland. It is the most commonly diagnosed cancer in all men with an estimated 180,000 new cases in 2016 (Siegel et al., 2016). This cancer type is also the second leading cause of cancer deaths in men, which primarily appears to be a result of late detection. Risk factors for developing this disease are advanced age, family history, and race.

Cannabinoids and their receptors have shown promise as potential therapeutics in prostate cancer. CB1 and CB2 receptor expression was shown to be elevated in prostate cancer compared to normal prostate tissue and are suggested to be positively correlated with poor disease outcome (Sarfaraz et al., 2005; Chung et al., 2009). Prostate cancer progression depends on androgen activity and while the ECS may influence this signaling, the interplay between these systems in the context of cancer is poorly understood (Sánchez et al., 2003a,b). Although numerous studies have evaluated the effect of cannabinoids on prostate cancer in cell culture studies, relatively few reports have determined if these effects occur in *in vivo* animal models. Di Marzo's group found that cannabis extracts enriched with CBD effectively decreased tumor growth in androgen receptor positive LNCaP xenografts but potentiated tumor growth of androgen receptor negative DU-145 xenografts (De et al., 2013). Furthermore, *in vitro* experiments using PC-3, DU-145, 22RV1, and LNCaP cell lines in this study found that CBD decreased androgen receptor expression suggesting that crosstalk between the CBD and androgen signaling pathways may affect tumor growth. CBD also significantly increased the generation of ROS and pro-apoptotic CHOP10 expression in these four prostate cancer cell lines (De et al., 2013). Similarly, JWH-015 led to decreased tumor proliferation in PC-3 derived xenografts. These anti-proliferative effects were examined *in vitro* and were suggested to be mediated by CB2 induced synthesis of ceramide (Olea-Herrero et al., 2009). While these studies show a promising role for cannabinoids in targeting prostate cancer, more research is needed to definitively establish cannabinoid efficacy *in vivo* and the importance of androgen receptor signaling in this system.

## Lung cancer

Lung cancer is the most deadly neoplasm in both males and females in the United States with a death toll anticipated to exceed 150,000 individuals in 2016 (Siegel et al., 2016). The two major types of lung cancer are small-cell lung cancer (SCLC) and non-small-cell lung cancer (NSCLC). Risk factors for developing this disease are tobacco smoking, genetic predisposition, and exposure to radon gas. Survival figures from this disease powerfully demonstrate the need for novel interventions. As such, cannabinoids have been evaluated *in vivo* for their efficacy against lung cancer.

CB1 receptors were expressed in 24% human NSCLC tissues, while CB2 receptor expression was found in 55% of NSCLC, suggesting a role for CB-receptors in tumor development (Preet et al., 2011). Cannabinoids such as Δ^9^-THC, CBD and Met-AEA elicited different effects in lung cancer animal models. CBD administration to mice with A549 cell xenografts decreased tumor growth, invasion and metastasis (Ramer et al., 2010a,b, 2012). Follow up *in vitro* experiments using A549, H358, and H460 lung cancer cell lines suggested that the anti-metastatic effect of CBD was mediated by upregulation of intracellular adhesion molecule-1 (ICAM-1), an anti-metastatic protein which has been suggested to suppress tumor growth via an immuno-surveillance mechanism (Wolfram et al., 2000; Ramer et al., 2012). In another study, CBD exhibited anti-tumor effects that were reversed by co-administration of a PPARγ antagonist *in vivo* (Ramer et al., 2013). Similarly, Preet et.al. found that the phytocannabinoid, Δ^9^-THC, downregulated Akt in A549-derived xenografts, which corresponded with decreased tumor growth and metastasis (Preet et al., 2008). In contrast, Zhu et al. showed that Δ^9^-THC increased the tumorigenicity of 3LL lung cancer cells in allografted immunocompetent mice and demonstrated that CB2 mediated inhibition of anti-tumor lymphocyte activity was the primary mechanism for accelerated tumor growth (Zhu et al., 2000). Furthermore, these investigators and the Arevalo group found that Δ^9^-THC administration had no effect on tumor growth in xenografted immunodeficient mice (Zhu et al., 2000; McKallip et al., 2005). Stable analogs of AEA also showed differential effects on lung cancer progression *in vivo*. The Dubinett group found that met-AEA increased tumor growth in a xenograft and allograft murine model and that COX-2 inhibition abrogated these effects (Gardner et al., 2003). Conversely, Bifulco et al. found that met-F-AEA treatment decreased tumor metastasis in a 3LL cell-derived xenograft model (Bifulco et al., 2001). These studies suggest that the ECS plays a role in both lung cancer progression and suppression. Consequently, several of these studies indicate that anti-tumor immunity is suppressed by certain cannabinoids, exacerbating tumor growth in immune-competent animals. As such, exploring the interplay between the immune system, the ECS and lung cancer development may be critical in determining whether cannabinoids are suitable for lung cancer treatment.

## Thyroid cancer

Thyroid cancer is the most frequently diagnosed endocrine malignancy in the United States. It is estimated that more than 64,000 new cases will be identified and 1900 deaths will result from this disease (Siegel et al., 2016). Although the death rate is relatively low compared to other types of cancer, therapeutic options are needed for aggressive forms of this disease whose lethality is thought to be associated with the differentiation state of the tumor (Lee et al., 2016). Relatively few studies have examined the effect of cannabinoids on thyroid tumor development *in vivo*, but these reports showed that the ECS was expressed and was capable of modulating tumor growth (Portella et al., 2003; Bifulco et al., 2004; Shi et al., 2008). For example, the activity of a variety of ECS modulators was examined in KiMol thyroid cells which generate undifferentiated tumors in xenograft models (Bifulco et al., 2004). In this study, the endogenously synthesized cannabinoid, 2-AG reduced thyroid tumor development. Similarly, agents that inhibited the EMT (VDM-11) and blocked AEA hydrolysis (AA-5HT) prevented *in vivo* tumor growth. Also, the metabolically stable molecules, arvanil and met-F-AEA also produced a significant reduction in tumor growth. The results of this study suggested that manipulation of the EC system was a viable option to prevent propagation of thyroid tumor cells. Additional preclinical studies were conducted with met-F-AEA. Using the xenograft model with KiMol cells it was determined that met-F-AEA prevented thyroid tumor growth and this that effect was reversed by antagonism of the CB1 receptor (Bifulco et al., 2001). Other studies with KiMol cells confirmed the anti-tumor activity of met-F-AEA and also indicated that this agent regulated tumor angiogenesis (Portella et al., 2003). Specifically, tumors isolated from met-F-AEA treated animals contained reduced levels of VEGF and VEGF receptor compared to vehicle treated animals. Furthermore, the reduction in VEGF signaling could be prevented by blockade of CB1 receptor activity. In another study, the synthetic cannabinoid, JWH-133, was tested in the highly aggressive ARO cell tumor model (Shi et al., 2008). Xenograft tumor growth using ARO/CB2 cells (a CB2 receptor overexpressing thyroid cancer cell line) was inhibited in mice treated with JWH-133 compared to vehicle. Cell culture experimentation suggested that JWH-133 toxicity was mediated by the CB2 receptor in contrast to met-F-AEA which was reported to initiate thyroid carcinoma cell death via CB1 (Portella et al., 2003). The data collected thus far suggest that synthetic EC system modulators represent novel therapeutic targets for thyroid cancer. However, because of the scarcity of *in vivo* thyroid cancer studies with cannabinoids, more extensive evaluation is needed to confidently define the role of the ECS in this malignancy.

## Skin cancer

The epidermis is comprised of different cell types including keratinocytes and melanocytes, which are the source of non-melanoma skin cancer (NMSC) and melanoma, respectively. Skin cancer is the most common cancer in the United States with approximately 5.4 million new lesions (3.3 million cases) of NMSC and more than 76,000 new cases of melanoma diagnosed each year (American Cancer Society, 2016; Siegel et al., 2016). Melanoma is more deadly and aggressive than NMSC. It is estimated that over 10,000 patients will die from melanoma in 2016 (Siegel et al., 2016). Several *in vitro* and *in vivo* studies suggest that the ECS plays a vital role in melanoma and NMSC development and progression (reviewed in Soliman et al., 2016a). It has been reported that CB1 and CB2 receptors were expressed in human melanoma cell lines as well as in human cutaneous melanoma biopsies (Blázquez et al., 2006). In addition, circulating levels of the endocannabinoid, 2-AG, increased in metastatic B16F10 mouse melanoma as well as in patients with metastatic melanoma suggesting a link between the ECS and melanoma progression (Sailler et al., 2014). Furthermore, in human and murine normal keratinocytes and non-melanoma skin tumors (e.g., papilloma, basal cell carcinoma, squamous cell carcinoma) CB1 and CB2 receptors were expressed and were required for the development of NMSC *in vivo* (Casanova et al., 2003; Zheng et al., 2008).

### Melanoma

The anti-tumor activity of phyto- and synthetic-cannabinoids against melanoma was reported in different *in vivo* studies using xenograft tumor models. Phytocannabinoid, Δ^9^-THC decreased proliferation, increased death and reduced the growth of CHL-1 melanoma xenografts (Armstrong et al., 2015). Using CB1/CB2-receptor deficient mice (CB1/2^−/−^), Glodde's group demonstrated that the anti-tumor effect of Δ^9^-THC on HCmel12 xenografts was dependent on cannabinoid receptors (Glodde et al., 2015). Synthetic cannabinoids, with different affinities for CB1 and CB2 receptors, have helped to shed light on the potential of targeting CB1 and CB2 receptors in melanoma. Mixed CB1/CB2 agonist, WIN-55212,2 decreased the progression and metastasis of B16F10 melanoma cells in mice after subcutaneous and intra-plantar implantation, respectively (Blázquez et al., 2006). Because the psychotropic effects of WIN-55212,2 are primarily mediated by the CB1 receptor, the anti-tumor effects of the non-psychoactive, CB2 selective cannabinoid, JWH-133 was examined. JWH-133 was as effective as WIN55212,2 in inhibiting melanoma tumor growth, suggesting that the anti-tumor activity of cannabinoids was mediated by CB2 receptor activation (Blázquez et al., 2006). Hence, the data generated thus far indicate that cannabinoid receptor agonists may be appropriate therapeutic targets for melanoma. Newer clinical approaches to combat melanoma involve mobilization of cytotoxic T-cells with monoclonal antibodies such as ipilimumab and nivolumab. Since cannabinoids are known immunomodulators, the effects of cannabinoids on cancer immunosurvailence must be addressed in future studies to have a complete understanding of the therapeutic potential of these molecules.

### Non-melanoma skin cancer

Several studies report that cannabinoids and endocannabinoids have anti-cancer activity against NMSC via cannabinoid receptor dependent or independent pathways (Van Dross et al., 2013; Soliman and Van Dross, 2015; Soliman et al., 2016b). There are two well-known animal models for NMSC, the UVB-light-induced skin carcinogenesis model (Fischer et al., 2007) or the two-stage chemical carcinogenesis protocol using a tumor initiator such as dimethylbenz[a]-anthracene (DMBA) and a tumor promoter such as 12-O-tetradecanoylphorbol-13-acetate (TPA) (Kiraly et al., 2016). Using CB1 and CB2 receptor deficient (CB1/2^−/−^) and wild type (CB1/2^+/+^) mice, it was determined that CB1 and CB2 receptors were required for papilloma formation initiated by UVB-light. In contrast, similar studies with CB1/2^+/+^ and CB1/2^−/−^ mice in the DMBA/TPA model demonstrated that cannabinoid receptors were not required for skin tumorigenesis. Data from these two different studies indicate that the involvement of ECS in skin cancer development is dependent on the type of the stimulus (Zheng et al., 2008; Gegotek et al., 2016). The *in vivo* anti-tumor activities of different synthetic cannabinoids have also been reported. WIN-55,212–2 and JWH-133 reduced the growth, impaired vascularization, and reduced the expression of various pro-angiogenic factors in the murine epidermal tumor cell PDV.C57 xenograft model (Casanova et al., 2003). In a different study, the anti-tumor activity of other synthetic cannabinoids was analyzed in the DMBA/TPA skin carcinogenesis model where JWH-018, JWH-122, and JWH-210 reduced tumor incidence and multiplicity compared to vehicle treated animals (Nakajima et al., 2013). Collectively, it appears that the ECS is involved in the formation of UVB-induced NMSC and that phyto- and synthetic- cannabinoids are capable of decreasing NMSC growth in different tumor models. It will be important for future investigations to include human NMSC tissue models (such as PDX) in the examination of cannabinoid activity since many of the existing reports primarily utilize mouse tissue xenograft and carcinogen-induced cancer models.

## Anti-cancer effects of cannabinoids in humans

Human studies that investigate the pharmacotherapeutic benefits associated with the use of cannabinoid ligands focus on reductions in pain, spasticity and cognitive deficits in a number of central and peripheral nervous system disorders (Velasco et al., 2016). To date only a few cannabis-based pharmacological agents are licensed for clinical use. In Europe, Sativex® is approved for treatment of spasticity associated with multiple sclerosis and in Canada it is additionally approved as an adjunct analgesic for cancer pain. The CB1 antagonist rimonabant (Acomplia®) was licensed for use in Europe to treat obesity and related conditions but was discontinued due to adverse effects (Fijal and Filip, 2016). In the United States and Europe, nabilone (Cesamet®) and dronabinol (Marinol®) are approved compounds for prevention of chemotherapy-induced nausea and vomiting. The emergence of preclinical studies that demonstrate the anti-tumor effects of cannabinoids are growing in number and have formed the basis of limited clinical studies which are beginning to shed light on the translational value of the preclinical work.

Guzman and colleagues were the first group to report a Phase I human study of the anti-metastasic effects of Δ^9^-THC. In this open-labeled two-part clinical trial, nine patients with recurrent glioblastoma who were refractory to surgery and radiotherapy were treated with intracranial Δ^9^-THC in combination with high-dose chemotherapeutic agent, temozolomide (Temodar®). Following a washout phase (a period in a clinical trial where patients receive no active medication), the safety and tolerability of Δ^9^-THC was then compared to placebo (Clinical trial ID# NCT01812603). The primary outcome measured was the incidence of adverse effects in patients receiving the combination of Δ^9^-THC and Temodar®. Secondary measures were the progression free survival at 6 months and overall survival. Additional study outcomes have not yet been reported. Δ^9^-THC was found to reduce tumor cell growth *in vitro* from 2 study participants and reduce tumor cell immunostaining in these same patients. Importantly, intracranial administration of Δ^9^-THC was found to be a safe and tolerable approach with no apparent psychoactive effects (Gúzman et al., 2006). In a 2011 case report by Foroughi et al. two children with septum pellucidum/forniceal policytic astrocytomas experienced tumor regression following craniotomies and partial excision. The tumor regression occurred during the time that cannabis was inhaled by the patients (Foroughi et al., 2011). In another study, a 14-year-old female Philadelphia chromosome positive patient who had been unsuccessfully treated with “traditional” therapy (i.e., chemotherapy, bone marrow transplant and radiation) for acute lymphoblastic leukemia experienced a dose-dependent management of the condition with orally administered cannabinoid extracts (Singh and Bali, 2013). A clear limitation of the current human studies evaluating the anti-cancer effects of cannabinoid compounds is the small patient size. To date, no study findings have been replicated in multiple cohorts. Moreover, the measured outcomes and study designs are often variable across trials, making it difficult to compare their findings. However, despite these challenges, the evidence generated in these human studies are still informative and, when taken together with the strong *in vivo* animal data demonstrating anti-tumor effects of cannabinoids, offer promise for a clinical role for cannabinoids in the eradication of tumors.

## Conclusion

To gain an understanding regarding the translational and therapeutic potential of cannabinoids, this review focused on examining the overall efficacy of these molecules in animal and human studies. The majority of *in vivo* animal studies discussed here indicate that cannabinoids from plant, synthetic and endogenous origin are capable of effectively decreasing tumor growth and invasion (Table 1). Furthermore, clinical studies evaluating cannabinoid efficacy in human subjects are limited, yet these studies showed that cannabinoids may be safe and effective anti-neoplastics. Because large scale clinical trials are lacking, examining the activity of cannabinoids in clinically translatable animal models (such as PDX) should be the goal of future research as this approach may provide a more accurate assessment of the therapeutic potential of cannabinoids.

This review also sought to couple *in vivo* animal studies with *in vitro* experiments in order to examine proposed cannabinoid mechanisms of action and to identify specific cellular targets. The studies reviewed herein indicate that cannabinoids elicit activity through cannabinoid receptor dependent and independent pathways. Moreover, processes such as ceramide production, ER-stress, autophagy, angiogenesis and matrix remodeling also appear to regulate the anti-tumor activity of cannabinoids. Hence, these investigations shed light on the role of cannabinoids on tumor growth *in vivo* and may ultimately pave the way for the development of novel cannabinoid therapeutics for cancer treatment.

## Author contributions

DL, ES, LG, and RV contributed to the conception and design of the study, the acquisition of data, and the analysis and interpretation of the data. All authors participated in drafting or revising the manuscript, and all authors approved the final version of the manuscript for submission.

### Conflict of interest statement

The authors declare that the research was conducted in the absence of any commercial or financial relationships that could be construed as a potential conflict of interest.

## Abbreviations

ACF: aberrant crypt foci
1-AG: 1- arachidonoyl glycerol
2-AG: 2-arachidonoyl glycerol
ABDH: alpha/beta-hydrolase domain; AEA arachidonoyl ethanolamide
Akt: protein kinase B
Ang2: angiopoietin-2 Protein
AOM: azoxymethane
BBB: blood-brain-barrier
CB1: cannabinoid receptor type 1; CB2 cannabinoid receptor type 2
CBD: cannabidiol
CBG: cannabigerol
CHOP10: C/EBP-homologous protein
COX-2: cyclooxygenase-2
CNS: central nervous system
CRC: colorectal cancer
DEN: diethylnitrosamine
DMBA: dimethylbenz[a]-anthracene
DNA: deoxyribonucleic acid
ECS: endocannabinoid system
EGFR: epidermal growth factor receptor
EMT: endocannabinoid membrane transporter
ER: estrogen receptors
ER-stress: endoplasmic reticulum stress
ERK: extracellular signal-regulated kinase
FAAH: fatty acid amide hydrolase
Fas: TNF receptor superfamily
GEMMs: genetically engineered mouse models
GFAP: glial fibrillary acidic protein
GPR55: G-protein coupled receptor 55
HCC: hepatocellular carcinoma
HER2: human epidermal growth factor receptor type 2
ICAM-1: intracellular adhesion molecule-1
Id-1: inhibitor of DNA binding protein
Jnk: c-jun N-terminal kinase
LC3: Microtubule-associated protein 1A/1B-light chain 3
MAGL: monoacylglycerol lipase
MEF: mouse embryo fibroblasts
MMP: matrix metalloproteinase
mTORC: mammalian target of rapamycin complex
NMSC: non-melanoma skin cancer
NSCLC: non-small-cell lung carcinoma
P-450: cytochrome P-450
pCXCR4: phospho-CXC chemokine receptor 4
PPARγ: peroxisome proliferator-activated receptor gamma
PR: progesterone receptor
p-S6: phospho-S6 ribosomal protein
ROS: reactive oxygen species
S-100B: S100 calcium-binding protein B
SCLC: small-cell lung carcinoma
THC: tetrahydrocannabinol
TIMP: tissue inhibitors of metalloproteinases
TMZ: temezolamide
TPA: 12-O-tetradecanoylphorbol-13-acetate
TNBC: triple negative breast cancer
TRB3: tribbles homolog protein 3
TRPM8: transient receptor potential cation channel, subfamily M, member 8
TRPV1: transient potential vanilloid receptor
UVB: ultraviolet light B
VEGF: vascular endothelial growth factor
VEGFR: vascular endothelial growth factor receptor.

## References

[B1] Aguado T. Carracedo A. Julien B. Velasco G. Milman G. Mechoulam R.. (2007). Cannabinoids induce glioma stem-like cell differentiation and inhibit gliomagenesis. J. Biol. Chem. 282, 6854–6862. 10.1074/jbc.M608900200 17202146

[B2] American Cancer Society (2016). Key Statistics for Basal and Squamous Cell Skin Cancers. Atlanta, GA: Online Source.

[B3] Armstrong J. L. Hill D. S. McKee C. S. Hernandez-Tiedra S. Lorente M. Lopez-Valero I.. (2015). Exploiting cannabinoid-induced cytotoxic autophagy to drive melanoma cell death. J. Invest. Dermatol. 135, 1629–1637. 10.1038/jid.2015.45 25674907

[B4] Aviello G. Romano B. Borrelli F. Capasso R. Gallo L. Piscitelli F.. (2012). Chemopreventive effect of the non-psychotropic phytocannabinoid cannabidiol on experimental colon cancer. J. Mol. Med. 90, 925–934. 10.1007/s00109-011-0856-x 22231745

[B5] Bifulco M. Laezza C. Portella G. Vitale M. Orlando P. De Petrocellis L. (2001). Control by the endogenous cannabinoid system of ras oncogene-dependent tumor growth. FASEB J. 15, 2745–2747. 10.1096/fj.01-0320fje 11687506

[B6] Bifulco M. Laezza C. Valenti M. Ligresti A. Portella G. DI Marzo V. (2004). A new strategy to block tumor growth by inhibiting endocannabinoid inactivation. FASEB J. 18, 1606–1608. 10.1096/fj.04-1754fje 15289448

[B7] Blázquez C. Carracedo A. Barrado L. Real P. J. Fernández-Luna J. L. Velasco G.. (2006). Cannabinoid receptors as novel targets for the treatment of melanoma. FASEB J. 20, 2633–2635. 10.1096/fj.06-6638fje 17065222

[B8] Blázquez C. Carracedo A. Salazar M. Lorente M. Egia A. González-Feria L.. (2008b). Down-regulation of tissue inhibitor of metalloproteinases-1 in gliomas: a new marker of cannabinoid antitumoral activity? Neuropharmacology 54, 235–243. 10.1016/j.neuropharm.2007.06.021 17675107

[B9] Blázquez C. Casanova M. L. Planas A. Gómez del P. T. Villanueva C. Fernández-Aceñero M. J.. (2003). Inhibition of tumor angiogenesis by cannabinoids. FASEB J. 17, 529–531. 10.1096/fj.02-0795fje 12514108

[B10] Blázquez C. González-Feria L. Alvarez L. Haro A. Casanova M. L. Guzmán M. (2004). Cannabinoids inhibit the vascular endothelial growth factor pathway in gliomas. Cancer Res. 64, 5617–5623. 10.1158/0008-5472.CAN-03-3927 15313899

[B11] Blázquez C. Salazar M. Carracedo A. Lorente M. Egia A. González-Feria L.. (2008a). Cannabinoids inhibit glioma cell invasion by down-regulating matrix metalloproteinase-2 expression. Cancer Res. 68, 1945–1952. 10.1158/0008-5472.CAN-07-5176 18339876

[B12] Borrelli F. Pagano E. Romano B. Panzera S. Maiello F. Coppola D.. (2014). Colon carcinogenesis is inhibited by the TRPM8 antagonist cannabigerol, a Cannabis-derived non-psychotropic cannabinoid. Carcinogenesis 35, 2787–2797. 10.1093/carcin/bgu205 25269802

[B13] Caffarel M. M. Andradas C. Mira E. Pérez-Gómez E. Cerutti C. Moreno-Bueno G.. (2010). Cannabinoids reduce ErbB2-driven breast cancer progression through Akt inhibition. Mol. Cancer. 9:196. 10.1186/1476-4598-9-196 20649976 PMC2917429

[B14] Caffarel M. M. Andradas C. Pérez-Gómez E. Guzmán M. Sanchez C. (2012). Cannabinoids: a new hope for breast cancer therapy? Cancer Treat. Rev. 38, 911–918. 10.1016/j.ctrv.2012.06.005 22776349

[B15] Caffarel M. M. Sarrio D. Palacios J. Guzmán M. Sánchez C. (2006). Delta9-tetrahydrocannabinol inhibits cell cycle progression in human breast cancer cells through Cdc2 regulation. Cancer Res. 66, 6615–6621. 10.1158/0008-5472.CAN-05-4566 16818634

[B16] Carracedo A. Gironella M. Lorente M. Garcia S. Guzmán M. Velasco G.. (2006). Cannabinoids induce apoptosis of pancreatic tumor cells via endoplasmic reticulum stress-related genes. Cancer Res. 66, 6748–6755. 10.1158/0008-5472.CAN-06-0169 16818650

[B17] Casanova M. L. Blázquez C. Martínez-Palacio J. Villanueva C. Fernández-Aceñero M. J. Huffman J. W.. (2003). Inhibition of skin tumor growth and angiogenesis *in vivo* by activation of cannabinoid receptors. J. Clin. Invest. 111, 43–50. 10.1172/JCI200316116 12511587 PMC151833

[B18] Chen L. Chen H. Li Y. Li L. Qiu Y. Ren J. (2015). Endocannabinoid and ceramide levels are altered in patients with colorectal cancer. Oncol. Rep. 34, 447–454. 10.3892/or.2015.3973 25975960

[B19] Chung S. C. Hammarsten P. Josefsson A. Stattin P. Granfors T. Egevad L.. (2009). A high cannabinoid CB(1) receptor immunoreactivity is associated with disease severity and outcome in prostate cancer. Eur. J. Cancer 45, 174–182. 10.1016/j.ejca.2008.10.010 19056257

[B20] Cianchi F. Papucci L. Schiavone N. Lulli M. Magnelli L. Vinci M. C.. (2008). Cannabinoid receptor activation induces apoptosis through tumor necrosis factor alpha-mediated ceramide de novo synthesis in colon cancer cells. Clin. Cancer Res. 14, 7691–7700. 10.1158/1078-0432.CCR-08-0799 19047095

[B21] De P. L. Ligresti A. Schiano M. A. Iappelli M. Verde R. Stott C. G.. (2013). Non-THC cannabinoids inhibit prostate carcinoma growth *in vitro* and *in vivo*: pro-apoptotic effects and underlying mechanisms. Br. J. Pharmacol. 168, 79–102. 10.1111/j.1476-5381.2012.02027.x 22594963 PMC3570006

[B22] DeMorrow S. Francis H. Gaudio E. Venter J. Franchitto A. Kopriva S.. (2008). The endocannabinoid anandamide inhibits cholangiocarcinoma growth via activation of the noncanonical Wnt signaling pathway. Am. J. Physiol. Gastrointest. Liver Physiol. 295, G1150–G1158. 10.1152/ajpgi.90455.2008 18832445 PMC2604798

[B23] Duntsch C. Divi M. K. Jones T. Zhou Q. Krishnamurthy M. Boehm P.. (2006). Safety and efficacy of a novel cannabinoid chemotherapeutic, KM-233, for the treatment of high-grade glioma. J. Neurooncol. 77, 143–152. 10.1007/s11060-005-9031-y 16314952

[B24] Elbaz M. Ahirwar D. Ravi J. Nasser M. W. Ganju R. K. (2016). Novel role of cannabinoid receptor 2 in inhibiting EGF/EGFR and IGF-I/IGF-IR pathways in breast cancer. Oncotarget. [Epub ahead of print]. 10.18632/oncotarget.9408 27213582 PMC5444694

[B25] Elbaz M. Nasser M. W. Ravi J. Wani N. A. Ahirwar D. K. Zhao H.. (2015). Modulation of the tumor microenvironment and inhibition of EGF/EGFR pathway: novel anti-tumor mechanisms of Cannabidiol in breast cancer. Mol. Oncol. 9, 906–919. 10.1016/j.molonc.2014.12.010 25660577 PMC4387115

[B26] Ellert-Miklaszewska A. Grajkowska W. Gabrusiewicz K. Kaminska B. Konarska L. (2007). Distinctive pattern of cannabinoid receptor type II (CB2) expression in adult and pediatric brain tumors. Brain Res. 1137, 161–169. 10.1016/j.brainres.2006.12.060 17239827

[B27] Falasca M. Ferro R. (2016). Role of the lysophosphatidylinositol/GPR55 axis in cancer. Adv. Biol. Regul. 60, 88–93. 10.1016/j.jbior.2015.10.003 26588872

[B28] Fijal K. Filip M. (2016). Clinical/therapeutic approaches for cannabinoid ligands in central and peripheral nervous system diseases: mini review. Clin. Neuropharmacol. 39, 94–101. 10.1097/WNF.0000000000000132 26818043

[B29] Fischer S. M. Pavone A. Mikulec C. Langenbach R. Rundhaug J. E. (2007). Cyclooxygenase-2 expression is critical for chronic UV-induced murine skin carcinogenesis. Mol. Carcinog. 46, 363–371. 10.1002/mc.20284 17219415 PMC2243235

[B30] Foroughi M. Hendson G. Sargent M. A. Steinbok P. (2011). Spontaneous regression of septum pellucidum/forniceal pilocytic astrocytomas–possible role of Cannabis inhalation. Childs. Nerv. Syst. 27, 671–679. 10.1007/s00381-011-1410-4 21336992

[B32] Galve-Roperh I. Sánchez C. Cortés M. L. Gómez del P. T. Izquierdo M. Guzman M. (2000). Anti-tumoral action of cannabinoids: involvement of sustained ceramide accumulation and extracellular signal-regulated kinase activation. Nat. Med. 6, 313–319. 10.1038/73171 10700234

[B33] Gardner B. Zhu L. X. Sharma S. Tashkin D. P. Dubinett S. M. (2003). Methanandamide increases COX-2 expression and tumor growth in murine lung cancer. FASEB J. 17, 2157–2159. 10.1096/fj.03-0254fje 12958151

[B34] Gegotek A. Biernacki M. Ambrozewicz E. Surazynski A. Wronski A. Skrzydlewska E. (2016). The cross-talk between electrophiles, antioxidant defence and the endocannabinoid system in fibroblasts and keratinocytes after UVA and UVB irradiation. J. Dermatol. Sci. 81, 107–117. 10.1016/j.jdermsci.2015.11.005 26674123

[B35] Glodde N. Jakobs M. Bald T. Tüting T. Gaffal E. (2015). Differential role of cannabinoids in the pathogenesis of skin cancer. Life Sci. 138, 35–40. 10.1016/j.lfs.2015.04.003 25921771

[B36] Grimaldi C. Pisanti S. Laezza C. Malfitano A. M. Santoro A. Vitale M.. (2006). Anandamide inhibits adhesion and migration of breast cancer cells. Exp. Cell Res. 312, 363–373. 10.1016/j.yexcr.2005.10.024 16343481

[B37] Gurley S. N. Abidi A. H. Allison P. Guan P. Duntsch C. Robertson J. H.. (2012). Mechanism of anti-glioma activity and *in vivo* efficacy of the cannabinoid ligand KM-233. J. Neurooncol. 110, 163–177. 10.1007/s11060-012-0958-5 22875710

[B38] Gúzman M. Duarte M. J. Blázquez C. Ravina J. Rosa M. C. Galve-Roperh I.. (2006). A pilot clinical study of Delta9-tetrahydrocannabinol in patients with recurrent glioblastoma multiforme. Br. J. Cancer. 95, 197–203. 10.1038/sj.bjc.6603236 16804518 PMC2360617

[B39] Huang L. Quinn M. A. Frampton G. A. Golden L. E. DeMorrow S. (2011a). Recent advances in the understanding of the role of the endocannabinoid system in liver diseases. Dig. Liver Dis. 43, 188–193. 10.1016/j.dld.2010.08.010 20934397 PMC3033442

[B40] Huang L. Ramirez J. C. Frampton G. A. Golden L. E. Quinn M. A. Pae H. Y.. (2011b). Anandamide exerts its antiproliferative actions on cholangiocarcinoma by activation of the GPR55 receptor. Lab. Invest. 91, 1007–1017. 10.1038/labinvest.2011.62 21464819 PMC3126905

[B42] Jin K. Teng L. Shen Y. He K. Xu Z. Li G. (2010). Patient-derived human tumour tissue xenografts in immunodeficient mice: a systematic review. Clin. Transl. Oncol. 12, 473–480. 10.1007/s12094-010-0540-6 20615824

[B43] Kaminska M. Ciszewski T. Lopacka-Szatan K. Miotla P. Staroslawska E. (2015). Breast cancer risk factors. Prz Menopauzalny 14, 196–202. 10.5114/pm.2015.54346 26528110 PMC4612558

[B44] Kargl J. Haybaeck J. Stancic A. Andersen L. Marsche G. Heinemann A.. (2013). O-1602, an atypical cannabinoid, inhibits tumor growth in colitis-associated colon cancer through multiple mechanisms. J. Mol. Med. 91, 449–458. 10.1007/s00109-012-0957-1 22965195 PMC3529923

[B45] Kiraly A. J. Soliman E. Jenkins A. Van Dross R. T. (2016). Apigenin inhibits COX-2, PGE2, and EP1 and also initiates terminal differentiation in the epidermis of tumor bearing mice. Prostaglandins Leukot. Essent. Fatty Acids. 104, 44–53. 10.1016/j.plefa.2015.11.006 26802941

[B46] Kociba R. J. Schwetz B. A. (1982). Toxicity of 2, 3, 7, 8-tetrachlorodibenzo-p-dioxin (TCDD). Drug Metab. Rev. 13, 387–406. 10.3109/03602538209029986 6213397

[B47] Kogan N. M. Blazquez C. Alvarez L. Gallily R. Schlesinger M. Guzman M.. (2006). A cannabinoid quinone inhibits angiogenesis by targeting vascular endothelial cells. Mol. Pharmacol. 70, 51–59. 10.1124/mol.105.021089 16571653

[B48] Kogan N. M. Rabinowitz R. Levi P. Gibson D. Sandor P. Schlesinger M.. (2004). Synthesis and antitumor activity of quinonoid derivatives of cannabinoids. J. Med. Chem. 47, 3800–3806. 10.1021/jm040042o 15239658

[B49] Kogan N. M. Schlesinger M. Peters M. Marincheva G. Beeri R. Mechoulam R. (2007). A cannabinoid anticancer quinone, HU-331, is more potent and less cardiotoxic than doxorubicin: a comparative *in vivo* study. J. Pharmacol. Exp. Ther. 322, 646–653. 10.1124/jpet.107.120865 17478614

[B50] Krishnamurthy M. Gurley S. Moore B. M. (2008). Exploring the substituent effects on a novel series of C1'-dimethyl-aryl Delta8-tetrahydrocannabinol analogs. Bioorg. Med. Chem. 16, 6489–6500. 10.1016/j.bmc.2008.05.034 18524604

[B51] Lee D. Y. Won J. K. Lee S. H. Park D. J. Jung K. C. Sung M. W.. (2016). Changes of clinicopathologic characteristics and survival outcomes of anaplastic and poorly differentiated thyroid carcinoma. Thyroid 26, 404–413. 10.1089/thy.2015.0316 26541309

[B52] Ligresti A. Moriello A. S. Starowicz K. Matias I. Pisanti S. De P. L.. (2006). Antitumor activity of plant cannabinoids with emphasis on the effect of cannabidiol on human breast carcinoma. J. Pharmacol. Exp. Ther. 318, 1375–1387. 10.1124/jpet.106.105247 16728591

[B53] Ling M. T. Wang X. Zhang X. Wong Y. C. (2006). The multiple roles of Id-1 in cancer progression. Differentiation 74, 481–487. 10.1111/j.1432-0436.2006.00083.x 17177845

[B54] Lukaszewicz-Zajac M. Mroczko B. Kornhuber J. Lewczuk P. (2014). Matrix metalloproteinases (MMPs) and their tissue inhibitors (TIMPs) in the tumors of central nervous system (CNS). J. Neural Transm. (Vienna). 121, 469–477. 10.1007/s00702-013-1143-5 24366530

[B56] Martínez-Martínez E. Gómez I. Martín P. Sánchez A. Román L. Tejerina E.. (2015). Cannabinoids receptor type 2, CB2, expression correlates with human colon cancer progression and predicts patient survival. Oncoscience 2, 131–141. 10.18632/oncoscience.119 25859556 PMC4381706

[B57] Massi P. Vaccani A. Ceruti S. Colombo A. Abbracchio M. P. Parolaro D. (2004). Antitumor effects of cannabidiol, a nonpsychoactive cannabinoid, on human glioma cell lines. J. Pharmacol. Exp. Ther. 308, 838–845. 10.1124/jpet.103.061002 14617682

[B58] McAllister S. D. Murase R. Christian R. T. Lau D. Zielinski A. J. Allison J.. (2011). Pathways mediating the effects of cannabidiol on the reduction of breast cancer cell proliferation, invasion, and metastasis. Breast Cancer Res. Treat. 129, 37–47. 10.1007/s10549-010-1177-4 20859676 PMC3410650

[B59] McKallip R. J. Nagarkatti M. Nagarkatti P. S. (2005). Delta-9-tetrahydrocannabinol enhances breast cancer growth and metastasis by suppression of the antitumor immune response. J. Immunol. 174, 3281–3289. 10.4049/jimmunol.174.6.3281 15749859

[B60] Michalski C. W. Oti F. E. Erkan M. Sauliunaite D. Bergmann F. Pacher P.. (2008). Cannabinoids in pancreatic cancer: correlation with survival and pain. Int. J. Cancer. 122, 742–750. 10.1002/ijc.23114 17943729 PMC2225529

[B61] Morales P. Blasco-Benito S. Andradas C. Gómez-Cañas M. Flores J. M. Goya P.. (2015). Selective, nontoxic CB(2) cannabinoid o-quinone with *in vivo* activity against triple-negative breast cancer. J. Med. Chem. 58, 2256–2264. 10.1021/acs.jmedchem.5b00078 25671648

[B62] Moreno E. Andradas C. Medrano M. Caffarel M. M. Pérez-Gómez E. Blasco-Benito S.. (2014). Targeting CB2-GPR55 receptor heteromers modulates cancer cell signaling. J. Biol. Chem. 289, 21960–21972. 10.1074/jbc.M114.561761 24942731 PMC4139213

[B63] Mukhopadhyay B. Schuebel K. Mukhopadhyay P. Cinar R. Godlewski G. Xiong K.. (2015). Cannabinoid receptor 1 promotes hepatocellular carcinoma initiation and progression through multiple mechanisms. Hepatology 61, 1615–1626. 10.1002/hep.27686 25580584 PMC4406817

[B64] Murase R. Kawamura R. Singer E. Pakdel A. Sarma P. Judkins J.. (2014). Targeting multiple cannabinoid anti-tumour pathways with a resorcinol derivative leads to inhibition of advanced stages of breast cancer. Br. J. Pharmacol. 171, 4464–4477. 10.1111/bph.12803 24910342 PMC4209152

[B66] Nakajima J. Nakae D. Yasukawa K. (2013). Structure-dependent inhibitory effects of synthetic cannabinoids against 12-O-tetradecanoylphorbol-13-acetate-induced inflammation and skin tumour promotion in mice. J. Pharm. Pharmacol. 65, 1223–1230. 10.1111/jphp.12082 23837590

[B67] Nasser M. W. Qamri Z. Deol Y. S. Smith D. Shilo K. Zou X.. (2011). Crosstalk between chemokine receptor CXCR4 and cannabinoid receptor CB2 in modulating breast cancer growth and invasion. PLoS ONE 6:e23901. 10.1371/journal.pone.0023901 21915267 PMC3168464

[B68] Olea-Herrero N. Vara D. Malagarie-Cazenave S. Díaz-Laviada I. (2009). Inhibition of human tumour prostate PC-3 cell growth by cannabinoids R(+)-Methanandamide and JWH-015: involvement of CB2. Br. J. Cancer. 101, 940–950. 10.1038/sj.bjc.6605248 19690545 PMC2743360

[B69] Ostrom Q. T. Gittleman H. Fulop J. Liu M. Blanda R. Kromer C.. (2015). CBTRUS statistical report: primary brain and central nervous system tumors diagnosed in the United States in 2008-2012. Neuro Oncol. 17(Suppl. 4), iv1–iv62. 10.1093/neuonc/nov189 26511214 PMC4623240

[B70] Pérez-Gómez E. Andradas C. Blasco-Benito S. Caffarel M. M. García-Taboada E. Villa-Morales M.. (2015). Role of cannabinoid receptor CB2 in HER2 pro-oncogenic signaling in breast cancer. J. Natl. Cancer Inst. 107:djv077. 10.1093/jnci/djv077 25855725

[B71] Peterson J. K. Houghton P. J. (2004). Integrating pharmacology and *in vivo* cancer models in preclinical and clinical drug development. Eur. J. Cancer 40, 837–844. 10.1016/j.ejca.2004.01.003 15120039

[B72] Portella G. Laezza C. Laccetti P. De Petrocellis L Di Marzo V Bifulco M. (2003). Inhibitory effects of cannabinoid CB1 receptor stimulation on tumor growth and metastatic spreading: actions on signals involved in angiogenesis and metastasis. FASEB J. 17, 1771–1773. 10.1096/fj.02-1129fje 12958205

[B73] Preet A. Ganju R. K. Groopman J. E. (2008). Delta9-Tetrahydrocannabinol inhibits epithelial growth factor-induced lung cancer cell migration *in vitro* as well as its growth and metastasis *in vivo*. Oncogene 27, 339–346. 10.1038/sj.onc.1210641 17621270

[B74] Preet A. Qamri Z. Nasser M. W. Prasad A. Shilo K. Zou X.. (2011). Cannabinoid receptors, CB1 and CB2, as novel targets for inhibition of non-small cell lung cancer growth and metastasis. Cancer Prev. Res. (Phila). 4, 65–75. 10.1158/1940-6207.CAPR-10-0181 21097714 PMC3025486

[B75] Qamri Z. Preet A. Nasser M. W. Bass C. E. Leone G. Barsky S. H.. (2009). Synthetic cannabinoid receptor agonists inhibit tumor growth and metastasis of breast cancer. Mol. Cancer Ther. 8, 3117–3129. 10.1158/1535-7163.MCT-09-0448 19887554 PMC4128286

[B76] Ramer R. Bublitz K. Freimuth N. Merkord J. Rohde H. Haustein M.. (2012). Cannabidiol inhibits lung cancer cell invasion and metastasis via intercellular adhesion molecule-1. FASEB J. 26, 1535–1548. 10.1096/fj.11-198184 22198381

[B77] Ramer R. Heinemann K. Merkord J. Rohde H. Salamon A. Linnebacher M.. (2013). COX-2 and PPAR-gamma confer cannabidiol-induced apoptosis of human lung cancer cells. Mol. Cancer Ther. 12, 69–82. 10.1158/1535-7163.MCT-12-0335 23220503

[B78] Ramer R. Merkord J. Rohde H. Hinz B. (2010b). Cannabidiol inhibits cancer cell invasion via upregulation of tissue inhibitor of matrix metalloproteinases-1. Biochem. Pharmacol. 79, 955–966. 10.1016/j.bcp.2009.11.007 19914218

[B79] Ramer R. Rohde A. Merkord J. Rohde H. Hinz B. (2010a). Decrease of plasminogen activator inhibitor-1 may contribute to the anti-invasive action of cannabidiol on human lung cancer cells. Pharm. Res. 27, 2162–2174. 10.1007/s11095-010-0219-2 20668920

[B80] Romano B. Borrelli F. Pagano E. Cascio M. G. Pertwee R. G. Izzo A. A. (2014). Inhibition of colon carcinogenesis by a standardized Cannabis sativa extract with high content of cannabidiol. Phytomedicine 21, 631–639. 10.1016/j.phymed.2013.11.006 24373545

[B81] Rosenberg D. W. Giardina C. Tanaka T. (2009). Mouse models for the study of colon carcinogenesis. Carcinogenesis 30, 183–196. 10.1093/carcin/bgn267 19037092 PMC2639048

[B82] Sailler S. Schmitz K. Jäger E. Ferreiros N. Wicker S. Zschiebsch K.. (2014). Regulation of circulating endocannabinoids associated with cancer and metastases in mice and humans. Oncoscience 1, 272–282. 10.18632/oncoscience.33 25594019 PMC4278301

[B83] Salazar M. Carracedo A. Salanueva I. J. Hernández-Tiedra S. Lorente M. Egia A.. (2009). Cannabinoid action induces autophagy-mediated cell death through stimulation of ER stress in human glioma cells. J. Clin. Invest. 119, 1359–1372. 10.1172/JCI37948 19425170 PMC2673842

[B84] Sánchez C. de Ceballos M. L. Gomez del P. T. Rueda D. Corbacho C. Velasco G.. (2001). Inhibition of glioma growth *in vivo* by selective activation of the CB(2) cannabinoid receptor. Cancer Res. 61, 5784–5789. 11479216

[B85] Sánchez M. G. Ruiz-Llorente L. Sánchez A. M. Díaz-Laviada I. (2003a). Activation of phosphoinositide 3-kinase/PKB pathway by CB(1) and CB(2) cannabinoid receptors expressed in prostate PC-3 cells. Involvement in Raf-1 stimulation and NGF induction. Cell. Signal. 15, 851–859. 10.1016/S0898-6568(03)00036-6 12834810

[B86] Sánchez M. G. Sánchez A. M. Ruiz-Llorente L. Díaz-Laviada I. (2003b). Enhancement of androgen receptor expression induced by (R)-methanandamide in prostate LNCaP cells. FEBS Lett. 555, 561–566. 10.1016/S0014-5793(03)01349-8 14675774

[B87] Sarfaraz S. Afaq F. Adhami V. M. Mukhtar H. (2005). Cannabinoid receptor as a novel target for the treatment of prostate cancer. Cancer Res. 65, 1635–1641. 10.1158/0008-5472.CAN-04-3410 15753356

[B88] Scott K. A. Dalgleish A. G. Liu W. M. (2014). The combination of cannabidiol and Delta9-tetrahydrocannabinol enhances the anticancer effects of radiation in an orthotopic murine glioma model. Mol. Cancer Ther. 13, 2955–2967. 10.1158/1535-7163.MCT-14-0402 25398831

[B89] Sharpless N. E. Depinho R. A. (2006). The mighty mouse: genetically engineered mouse models in cancer drug development. Nat. Rev. Drug Discov. 5, 741–754. 10.1038/nrd2110 16915232

[B90] Shi Y. Zou M. Baitei E. Y. Alzahrani A. S. Parhar R. S. Al-Makhalafi Z.. (2008). Cannabinoid 2 receptor induction by IL-12 and its potential as a therapeutic target for the treatment of anaplastic thyroid carcinoma. Cancer Gene Ther. 15, 101–107. 10.1038/sj.cgt.7701101 18197164

[B91] Siegel R. L. Miller K. D. Jemal A. (2016). Cancer statistics, 2016. CA Cancer J. Clin. 66, 7–30. 10.3322/caac.21332 26742998

[B92] Singer E. Judkins J. Salomonis N. Matlaf L. Soteropoulos P. McAllister S.. (2015). Reactive oxygen species-mediated therapeutic response and resistance in glioblastoma. Cell Death Dis. 6:e1601. 10.1038/cddis.2014.566 25590811 PMC4669764

[B93] Singh S. K. Hawkins C. Clarke I. D. Squire J. A. Bayani J. Hide T.. (2004). Identification of human brain tumour initiating cells. Nature 432, 396–401. 10.1038/nature03128 15549107

[B94] Singh Y. Bali C. (2013). Cannabis extract treatment for terminal acute lymphoblastic leukemia with a Philadelphia chromosome mutation. Case Rep. Oncol. 6, 585–592. 10.1159/000356446 24474921 PMC3901602

[B95] Soliman E. Henderson K. L. Danell A. S. Van Dross R. (2016b). Arachidonoyl-ethanolamide activates endoplasmic reticulum stress-apoptosis in tumorigenic keratinocytes: role of cyclooxygenase-2 and novel J-series prostamides. Mol. Carcinog. 55, 117–130. 10.1002/mc.22257 25557612

[B96] Soliman E. Ladin D. A. Van Dross R. (2016a). Cannabinoids as therapeutics for non-melanoma and melanoma skin cancer. J. Dermatol. Clin. Res. 4, 1069–1076. Available online at: http://www.jscimedcentral.com/Dermatology/dermatology-4-1069.pdf

[B97] Soliman E. Van Dross R. (2015). Anandamide-induced endoplasmic reticulum stress and apoptosis are mediated by oxidative stress in non-melanoma skin cancer: receptor-independent endocannabinoid signaling. Mol. Carcinog. [Epub ahead of print]. 10.1002/mc.22429 26513129

[B98] Soroceanu L. Murase R. Limbad C. Singer E. Allison J. Adrados I.. (2013). Id-1 is a key transcriptional regulator of glioblastoma aggressiveness and a novel therapeutic target. Cancer Res. 73, 1559–1569. 10.1158/0008-5472.CAN-12-1943 23243024 PMC3594064

[B99] Tanasescu R. Constantinescu C. S. (2010). Cannabinoids and the immune system: an overview. Immunobiology 215, 588–597. 10.1016/j.imbio.2009.12.005 20153077

[B100] Teicher B. A. (2006). Tumor models for efficacy determination. Mol. Cancer Ther. 5, 2435–2443. 10.1158/1535-7163.MCT-06-0391 17041086

[B101] Torres S. Lorente M. Rodríguez-Fornés F. Hernández-Tiedra S. Salazar M. García-Taboada E.. (2011). A combined preclinical therapy of cannabinoids and temozolomide against glioma. Mol. Cancer Ther. 10, 90–103. 10.1158/1535-7163.MCT-10-0688 21220494

[B102] Van Dross R. Soliman E. Jha S. Johnson T. Mukhopadhyay S. (2013). Receptor-dependent and receptor-independent endocannabinoid signaling: a therapeutic target for regulation of cancer growth. Life Sci. 92, 463–466. 10.1016/j.lfs.2012.09.025 23069587 PMC4226396

[B103] Vara D. Morell C. Rodríguez-Henche N. Diaz-Laviada I. (2013). Involvement of PPARgamma in the antitumoral action of cannabinoids on hepatocellular carcinoma. Cell Death Dis. 4:e618. 10.1038/cddis.2013.141 23640460 PMC3674350

[B104] Vara D. Salazar M. Olea-Herrero N. Guzmán M. Velasco G. Diaz-Laviada I. (2011). Anti-tumoral action of cannabinoids on hepatocellular carcinoma: role of AMPK-dependent activation of autophagy. Cell Death Differ. 18, 1099–1111. 10.1038/cdd.2011.32 21475304 PMC3131949

[B105] Velasco G. Hernández-Tiedra S. Dávila D. Lorente M. (2016). The use of cannabinoids as anticancer agents. Prog. Neuropsychopharmacol. Biol. Psychiatry. 64, 259–266. 10.1016/j.pnpbp.2015.05.010 26071989

[B106] Wan F. Herold-Mende C. Campos B. Centner F. S. Dictus C. Becker N.. (2011). Association of stem cell-related markers and survival in astrocytic gliomas. Biomarkers 16, 136–143. 10.3109/1354750X.2010.536256 21323603

[B107] Wolfram R. M. Budinsky A. C. Brodowicz T. Kubista M. Köstler W. J. Kichler-Lakomy C.. (2000). Defective antigen presentation resulting from impaired expression of costimulatory molecules in breast cancer. Int. J. Cancer. 88, 239–244. 10.1002/1097-0215(20001015)88:2<239::AID-IJC15>3.0.CO;2-Z 11004675

[B108] Xu X. Liu Y. Huang S. Liu G. Xie C. Zhou J.. (2006). Overexpression of cannabinoid receptors CB1 and CB2 correlates with improved prognosis of patients with hepatocellular carcinoma. Cancer Genet. Cytogenet. 171, 31–38. 10.1016/j.cancergencyto.2006.06.014 17074588

[B109] Zheng D. Bode A. M. Zhao Q. Cho Y. Y. Zhu F. Ma W. Y.. (2008). The cannabinoid receptors are required for ultraviolet-induced inflammation and skin cancer development. Cancer Res. 68, 3992–3998. 10.1158/0008-5472.CAN-07-6594 18483286 PMC2390870

[B110] Zhu L. X. Sharma S. Stolina M. Gardner B. Roth M. D. Tashkin D. P.. (2000). Delta-9-tetrahydrocannabinol inhibits antitumor immunity by a CB2 receptor-mediated, cytokine-dependent pathway. J. Immunol. 165, 373–380. 10.4049/jimmunol.165.1.373 10861074

[B111] Zhu P. J. (2006). Endocannabinoid signaling and synaptic plasticity in the brain. Crit. Rev. Neurobiol. 18, 113–124. 10.1615/CritRevNeurobiol.v18.i1-2.120 17725514

