# Corrigendum: Preclinical and Clinical Assessment of Cannabinoids as Anti-Cancer Agents

**DOI:** 10.3389/fphar.2021.732903

**Published:** 2021-07-16

**Authors:** Daniel A. Ladin, Eman Soliman, LaToya Griffin, Rukiyah Van Dross

**Affiliations:** ^1^ Department of Pharmacology and Toxicology, Brody School of Medicine, East Carolina University, Greenville, NC, United States; ^2^ Department of Pharmacology and Toxicology, Faculty of Pharmacy, Zagazig University, Zagazig, Egypt; ^3^ Center for Health Disparities, East Carolina University, Greenville, NC, United States

**Keywords:** cannabinoids, cancer, *in vivo*, clinical studies, clinical trials, therapeutics, cancer models, endocannabinoids

In the original article, the **Reference** for “Blazquez, C. et al. (2006)” was incorrectly written as “Naeem et al. (2006).” The correct and full citation is “Blázquez, C., Carracedo, A., Barrado, L., Real, P. J., Fernández-Luna, J. L., Velasco, G., Malumbres, M., Guzmán, M. (2006). Cannabinoid receptors as novel targets for the treatment of melanoma. FASEB J. 20, 2633–2635. 10.1096/fj.06-6638fje.”

In the original article, the **Reference** for “Velasco, G., (2016)” was incorrectly written as “Fujimura et al. (1989).” The correct and full citation is “Velasco, G., Hernandez-Tiedra, S., Davila, D., and Lorente, M. (2016). The use of cannabinoids as anticancer agents. Prog. Neuropsychopharmacol. Biol. Psychiarty. 64, 259-266. 10.1016/j.pnpbp 2015.05.010.”

In the original article, the **Reference** for “Salazar (2009)” was incorrectly written as “Jenkins, M.A., et al. (2006).” The correct and full citation is “Salazar, M., Carracedo, A., Salanueva, I. J., Hernandez-Tiedra, S., Lorente, M., Egia, A., Vazquez, P., Blazquez, C., Torres, S., Garcia, S., Nowak, J., Fimai, G. M., Piacentini, M., Cecconi, F., Pandolfi, P. P., Gonzalez-Feria, L., Iovanna, J. L., Guzman, M., Boya, P., and Velasco, G. (2009). Cannabinoid action induces autophagy-mediated cell death through stimulation of ER stress in human glioma cells. J. Clin. Invest. 119, 1359-1372. 10.1172/JCI37948.”

In the original article, the **Reference** for “McKallip (2005)” was incorrectly written as “Martin-Banderas et al. (2015).” The correct and full citation is “McKallip, R. J., Nagarkatti, M., and Nagarkatti, P. S. (2005). Delta-9- tetrahydrocannabinol enhances breast cancer growth and metastasis by suppression of the antitumor immune response. J. Immunol 174, 3281-3289. 10.4049/jimmunol.174.6.3281.”

The authors apologize for these errors and state that this does not change the scientific conclusions of the article in any way. The original article has been updated.

